# Genetics of the human Y chromosome and its association with male infertility

**DOI:** 10.1186/s12958-018-0330-5

**Published:** 2018-02-17

**Authors:** Stacy Colaco, Deepak Modi

**Affiliations:** Department of Molecular and Cellular Biology, ICMR-National Institute for Research in Reproductive Health, JM Street, Parel, Mumbai, Maharashtra 400012 India

**Keywords:** Infertility, Y chromosome, Microdeletions, AZF, AZFc, gr/gr deletions, Prevalence, Spermatogenesis

## Abstract

The human Y chromosome harbors genes that are responsible for testis development and also for initiation and maintenance of spermatogenesis in adulthood. The long arm of the Y chromosome (Yq) contains many ampliconic and palindromic sequences making it predisposed to self-recombination during spermatogenesis and hence susceptible to intra-chromosomal deletions. Such deletions lead to copy number variation in genes of the Y chromosome resulting in male infertility. Three common Yq deletions that recur in infertile males are termed as AZF (Azoospermia Factor) microdeletions viz. AZFa, AZFb and AZFc. As estimated from data of nearly 40,000 Y chromosomes, the global prevalence of Yq microdeletions is 7.5% in infertile males; however the European infertile men are less susceptible to Yq microdeletions, the highest prevalence is in Americans and East Asian infertile men. In addition, partial deletions of the AZFc locus have been associated with infertility but the effect seems to be ethnicity dependent. Analysis of > 17,000 Y chromosomes from fertile and infertile men has revealed an association of gr/gr deletion with male infertility in Caucasians and Mongolian men, while the b2/b3 deletion is associated with male infertility in African and Dravidian men. Clinically, the screening for Yq microdeletions would aid the clinician in determining the cause of male infertility and decide a rational management strategy for the patient. As these deletions are transmitted to 100% of male offspring born through assisted reproduction, testing of Yq deletions will allow the couples to make an informed choice regarding the perpetuation of male infertility in future generations. With the emerging data on association of Yq deletions with testicular cancers and neuropsychiatric conditions long term follow-up data is urgently needed for infertile men harboring Yq deletions. If found so, the information will change the current the perspective of androgenetics from infertility and might have broad implication in men health.

**Electronic supplementary material:**

The online version of this article (10.1186/s12958-018-0330-5) contains supplementary material, which is available to authorized users.

## Background

Human spermatogenesis is an intricate biological process that begins with the mitotic division of spermatogonia to give rise to primary spermatocytes which in turn undergo the first meiotic division to form secondary spermatocytes. After a second meiosis cycle, these secondary spermatocytes produce haploid cells called round spermatids, which subsequently form elongated spermatids which finally differentiate into mature spermatozoa. The spermatogenic process relies on the concerted actions of various hormones, local secretory factors and testis-specific genes. Defects at any of these levels can lead to accumulation of errors resulting in impaired spermatogenesis leading to male infertility. According to the World Health Organization [1], male infertility refers to the inability of the male partner to cause pregnancy in a clinically normal female. Almost 30 million males worldwide are infertile with the largest niches of male infertility occurring in central and Eastern Europe [8–12%] and Australia [8–9%] [2]. As per WHO 2010 male infertility can be classified based on seminogram under following categories:

- Azoospermia- Absence of sperm in the ejaculate, it can be classified as obstructive azoospermia [OA] where absence of sperm in the ejaculate is observed as a result of problems in sperm delivery or non-obstructive azoospermia [NOA] where there is absence of sperm in the semen due to abnormal sperm production. NOA constitutes 60% of all cases of azoospermia
- Oligozoospermia- Less than 15–20 × 10^6^ spermatozoa in the ejaculate
- Severe oligozoospermia- Less than 5 × 10^6^ spermatozoa in the ejaculate
- Normozoospermia- Normal values of sperms in the ejaculate
- Asthenozoospermia- Low levels of motility observed in less than 50% of sperms
- Teratozoospermia- Less than 30% of sperms have normal morphology
- Aspermia- Failure in ejaculating semen

## Causes of male infertility

Male infertility can be attributed to several factors such as cryptorchidism [absence of one or both testes in the scrotum], varicocele [abnormal enlargement of the pampiniform venous plexus in the scrotum], endocrinological disorders, obstruction/absence of seminal pathways, infections, alcohol consumption or chemotherapy [3, 4]. However, genetic alterations have also emerged as one of the leading cause of male infertility. Genetic defects commonly observed in infertile males include karyotypic abnormalities, gene copy number variations [CNVs], single gene mutations/polymorphisms and deletions on the long arm of the Y chromosome [Yq microdeletions]. These genetic defects impede the development of the male gonads or urogenital tract during development, cause arrest of germ cell production and/or maturation or produce non-functional spermatozoa. Amongst the various factors, karyotypic abnormalities and Yq microdeletions are the leading genetic causes of male infertility. In this review, we present the current knowledge of the human Y chromosome, its genes and how the defects in these genes lead to male infertility.

## The human Y chromosome

**Fig. 1:**
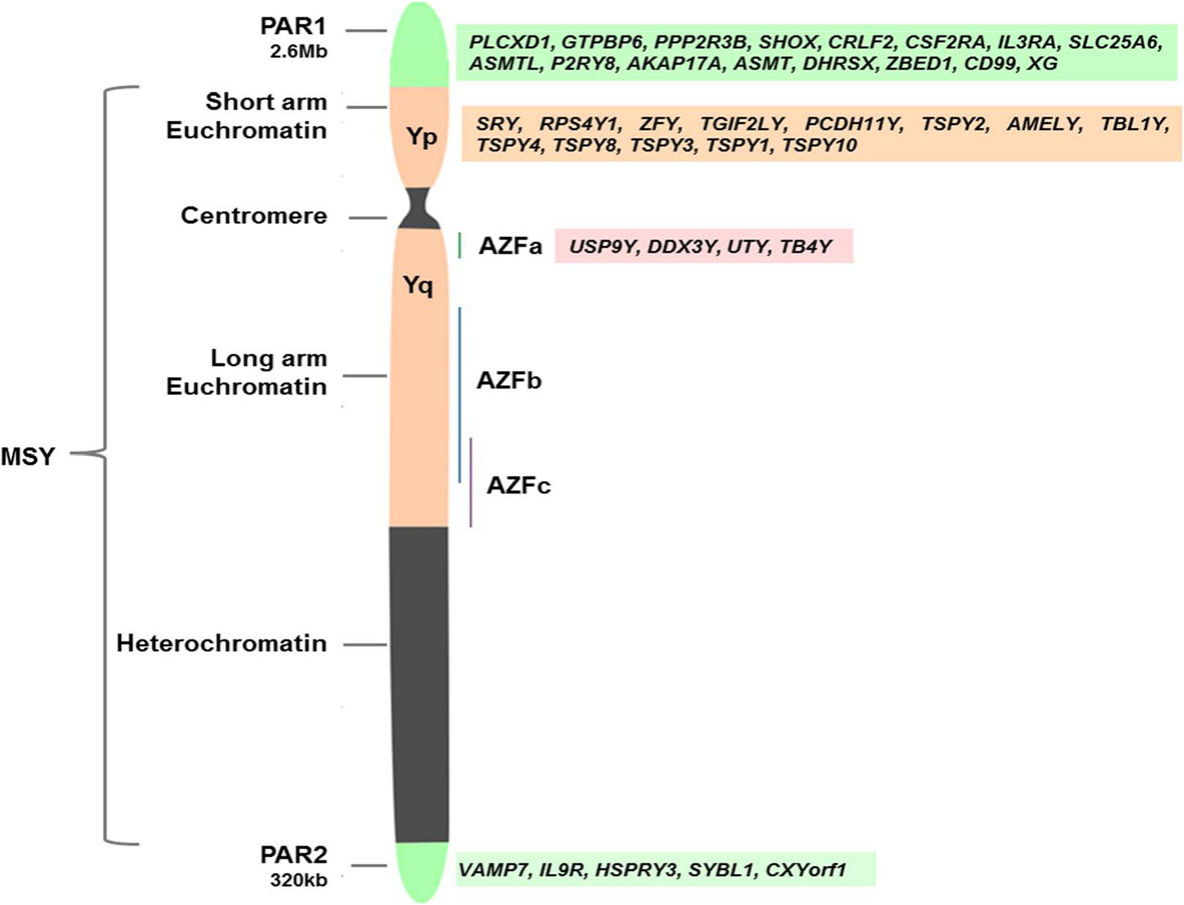
Structure of the human Y chromosome. The Pseudo Autosomal Regions [PAR1 and PAR2] are located at the terminal ends of the Y chromosome. The green boxes show the genes encoded in these regions. Yp is the short arm of the Y chromosome and the genes within it are show in the peach box. The long arm, Yq, is composed of both euchromatin and the genetically inactive heterochromatin regions. This region contains the Azoospermia factors AZFa, AZFb and AZFc. The pink box shows the genes in the AZFa region. The heterochromatin is not known to harbor any known genes. The region beyond the PAR is termed as Male Specific Region on Y (MSY)

Mammalian sex chromosomes evolved from autosomes at least 180 million years ago. The first step in differentiation of the Y chromosome involved the acquisition of the testis-determining gene followed by large-scale inversions and sequential suppression of recombination between the X and Y chromosomes in a stepwise fashion [5–7]. A detailed overview on the evolution of the human Y chromosome and its present day status has been a subject of recent reviews [5]. Cytogenetically, the human Y is an acrocentric chromosome composed of two pseudoautosomal regions (PARs), a short arm (Yp) and the long arm (Yq) that are separated by a centromere (Fig. 1). While the PARs and the short arm are euchromatic, a large portion of the long arm is heterochromatic with the exception of the proximal portion juxtaposed to the centromere which is euchromatic in nature.

## The pseudo autosomal regions (PARs)

The PAR1 and PAR2 of the Y chromosome are short regions of homology between the mammalian X and Y chromosomes; the PAR1 is located on the tip of the p arm and the PAR2 on the tip of the q arm (Fig. 1). Due to the diversity in the genetic sequences of the X and Y chromosomes, they do not undergo pairing during meiosis, except for the regions in PAR which pair and recombine with the PARs in the X chromosome during meiosis. However, the recombination and pairing of PARs is temporally and genetically distinct from that of the rest of the genome [8]. The PARs have a delayed double stranded break (DSBs) formation and pairing as compared to autosomes due to the fact that the PARs initiate DSB formation only after all the autosomal DSBs have been repaired.

Despite this delayed recombination, the cross over rate in PAR1 occurs more rapidly than it does on the autosomes [9]. In mice, on average, one meiotic DSB forms every 10 mega base pairs [Mb] while in the PARs it spans a mere 0.7 Mb [10]. It is proposed that in the PARs, deoxyribonucleic acid [DNA] is packaged into several small loops as compared to fewer but larger loops in the autosomes, thus allowing greater DSB formation in the PARs. Indeed, reports indicate that PAR1 contains several recombination hotspots, which have activities that differ significantly among human populations [11]. The genetic control of DSB formation in PARs is also distinct from that of autosomes. Autosomal pairing is dependent on SPO11βm while the X-Y paring is SPO11α dependent. It has been observed that PAR DSBs are not formed in mice that lack SPO11α leading to X-Y mis-segregation and male infertility, the autosomal DSBs are somehow not majorly affected [8]. Interestingly, studies have also associated deletion of human PAR1 with total male sterility [12, 13], reduced recombination in PAR1 has been associated with increased frequency of sex chromosome aneuploidy in sperm, leading to X-chromosome monosomy (Turner syndrome) or XXY (Kleinfelter syndrome) in the offspring [14, 15]. These observations suggest that although the Y chromosome PARs behave like autosomes, the mechanisms by which they do  so is different and also has a distinct genetic control.

## Genes in PAR1 and PAR2

While the PARs have distinct genetic control as compared to autosomes, wide differences also exist between the two PARs in terms of their genetic content and their functions. Together the PARs contain at least 29 genes, with diverse roles in cell signalling, transcriptional regulation and mitochondrial function [16]. PAR1 contains genes, all of which are known to escape X inactivation (Fig. 1). Interestingly, defects in genes of the PAR1 are associated with mental and stature disorders. Loss of short stature homeobox [*SHOX*] gene in the PAR1 is related to short stature of Turner syndrome [17]; mutation in *SHOX* are reported in patients with idiopathic growth retardation [18]. PAR1 loci are also reported to be associated with schizophrenia and bipolar disorder [19, 20].

PAR2 on the other hand is a much shorter region, spanning only 320 kilobase pairs [kb]. Unlike in the PAR1, crossovers in PAR2 occur at a rate similar to the genome average, in both sexes, suggesting behaviour similar to many autosomal regions. PAR is not necessary for fertility [21]. Unlike PAR1 which is gene rich, the PAR2 region contains only five genes of which two, *HSPRY3* and *SYBL1*, are inactivated on Y chromosome and subjected to X inactivation in females [22]. Additional file 1: Table S2. lists the different 226 genes on the Y chromosome, their cellular expression 227 in testis, putative function and role in spermatogenesis.

Beyond the PAR1 and PAR2, reports also suggest the existence of a 3.5 Mb region termed as PAR3. This region is said to have originated at Xq21.3 when a 3.5 Mb region of the X chromosome underwent duplication and transposition on the Y chromosome at Yp11.2 approximately 5–6 million years ago [23]. It is reported that Yp11.2 and Xq21.3 have 98.78% identity and a high concentration of tandem repeats. Interestingly, allelic unequal recombination also occurs between the two X transposed regions [24]. This PAR3 region is however found only in 2% of the general population and the functional significance (if any) of this PAR is unknown.

## The non-recombining region of Y (NRY)

NRY is defined as the locus beyond the PARs which does not undergo recombination during meiosis due to lack of homology with the X chromosome. Cytogenetically this region is divided into two regions viz. the heterochromatic and the euchromatic regions. The heterochromatic region of the Y chromosome comprises distal Yq that contains two highly repetitive sequences families, DYZ1 and DYZ2 [25]. Variation in the size of the Y chromosome long arm heterochromatin within an individual have been reported [26] however its clinical significance remains unknown.

The euchromatic region encompasses the para-centromeric region and the short and long arm of the Y. This region is also referred to as male specific region on Y (MSY) and was for a long time thought to be a functional wasteland. However research in the last two decades have revealed important roles of the euchromatic region ranging from sex determination to regulation of brain functions. Discussed below is our current understanding of the human MSY and alterations in this region that affect human health.

## Euchromatic region of Y

The euchromatic region of the Y lies distal to the PAR1 and consists of the short arm para-centromeric region, the centromere and the long arm para-centromeric region (Fig. 1). This region contains sequences that are subdivided into three discrete classes: X-transposed, X-degenerate and ampliconic [27]. The X-transposed sequences are so named, because of a massive X to Y transposition that occurred about 3–4 million years ago. Most of these sequences are composed of repeat elements such as Alu, retroviral and Long interspersed nuclear elements [LINE1]. Some of the genes belonging to this region have ubiquitous tissue expression; the ampliconic sequences contain genes and transcription units that are expressed solely in the testes [28]. The protein products of the MSY genes, contribute to gonad formation, regulation of spermatogenesis, brain, heart, and kidney development [29, 30] suggesting its critical functions in tissue development and its adult functions. Approximately 70 genes have been identified on the Y chromosome and described below are some of these genes. Additional file 1: Table S2 enlists the different genes on the Y chromosome along with their expression, function and role in spermatogenesis.

## Genes on the short arm of the Y chromosome [Yp]

### Sex determining region on Y [*SRY*]

In the year 1959 two scientific reports on the Klinefelter syndrome and on the Turner syndrome [31, 32], described for the very first time that the human Y chromosome contained at least one sex-determining gene that was responsible for the maleness of the embryo. A large numbers of sex reversed patients were subsequently identified to have deletions in portions of the Yp (XY sex reversal) or had additional portions of the Yp (XX males). These patients immensely contributed to the discovery of the SRY gene which was responsible for testis determination during embryogenesis. In 1990, the gene responsible for testicular determination, *SRY* (Sex-determining Region on the Y chromosome), was identified [33, 34] and was found to be located on the short arm of the Y chromosome close to the pseudoautosomal boundary. This gene is thought to have been evolved by a mutation in the *SOX3* gene. The human *SRY* is a single exon that encodes a protein of 204 amino acids which contains a conserved DNA-binding domain. *SRY* is essential for initiating testis development and differentiation of the bi-potential gonad into Sertoli cells, which then support differentiation and development of the male germline. Hence this gene has been proposed to be the master gene regulating the cascade of testis determination [35]. Mutations in the SRY gene are identified in approximately 15% 46, XY females (Swyer syndrome); translocation of the *SRY* gene to the X chromosome is reported in a subset of 46, XX males [36]. Beyond its expression in developing testis, *SRY* is reportedly expressed in adult testis and even ejaculated spermatozoa [37] the functional significance of which is yet unclear. In addition *SRY* is also expressed in other somatic tissues such as adipose, oesophagus, thymus, adrenal glands, brain kidneys and also in some cancer cell lines [28, 29] suggesting its functions beyond sex determination.

## Zinc Finger protein, Y linked [*ZFY*]

Another gene on the p arm of the Y chromosome is *ZFY* which encodes a zinc finger-containing protein and functions as a transcription factor. Expressed in almost all somatic tissues [28, 29], it is proposed to play a role in spermatogenesis, particularly in promoting meiotic division and sperm formation [38–41] Refer Additional file 1: Table S2. While mice knockout for Zfy genes are infertile [42], despite its expression in multiple tissues, a rare deletion of *ZFY* and *SRY* in a woman was not associated with Turner syndrome stigmata. So far, no mutations of *ZFY* have been reported, indicating that *ZFY* may not have any critical somatic functions [43].

## Amelogenin, Y linked [*AMELY*]

The *AMELY*, encodes a member of the amelogenin family of extracellular matrix proteins which are involved in biomineralization during tooth enamel development. This gene has a paralogoue on the X chromosome, *AMELX* and a mutation in this gene leads to amelogenesis imperfecta. *AMELY* is expressed at only 10% of the level of *AMELX* and the amelogenin paralogoues, there exist men with deletion of *AMELY*, but have no apparent phenotype [44], suggesting that absence of *AMELY* has no major deleterious effects.

## Transducin Beta-like 1Y [*TBL1Y*]

The Transducin Beta-like 1Y (*TBL1Y*), is a Y-linked homologue of *TBL1X* that is related with X-linked late-onset sensorineural deafness. *TBLR1*, a homologue of *TBL1Y*, and *TBL1X* act as a co-repressor/co-activator for several nuclear receptors and transcription factors. A recent study [45] has demonstrated differential expression of the *TBL1Y* during cardiac differentiation of human embryonic stem cells. Interestingly it was noted that the *TBL1Y* protein showed a significant increase during differentiation while the expression level of *TBL1X* simultaneously decreased. When the cellular levels of *TBL1Y* decreased, the authors observed reduced rates of cardiac differentiation as well as an increase in the probability of impaired contractions suggesting that *TBL1Y* knockdown may have negatively impacted cardiogenesis. Another study [46] has reported that the *TBL1Y(A)-USP9Y(A)* haplotype of the Y chromosome, present only in black people of African origin, contributed to a favourable lipoprotein pattern that most likely contributed to their reduced susceptibility to coronary heart disease. The role of *TBL1Y* in testis [if any] remains unknown.

## Protocadherin 11, Y linked [*PCDH11Y*]

Another gene of the p arm of the human Y chromosome is, *PCDH11Y*. This gene has an homologue on the *PCDH11X*; is a protocadherin and expressed in multiple tissues including the testis and brain [28, 29, 47]. It is proposed to play a role in cell-cell recognition during brain development and establishment of cerebral asymmetries in humans [48]. Deletions of both the X and Y *PCDH11* are associated with language delay [49].

## Testis-specific protein Y-linked [*TSPY*]

The Yp has an array of genes encoding the testis-specific protein Y linked (*TSPY*) which also has an X-homologue, *TSPX*. These proteins function as a proto-oncogene and a tumour suppressor respectively and are also cell cycle regulators [50]. Expressed in a variety of tissue including cancers, *TSPY* is a candidate gene for gonadoblastoma and variations in *TSPY* genes are associated with compromised spermatogenesis [51, 52] although a recent study has refuted this view [53]. For more information on the genes on the p arm of the Y chromosome refer to Additional file 1: Table S2.

## Genes on the long arm of the Y chromosome [Yq]

While the short arm of the Y chromosome was yet assumed to have some transcribing genes, the long arm of the Y chromosome was believed to be genetically inert. This myth was disproved in 1997 when 12 novel genes or gene families with ten full-length complementary DNA sequence were identified in the human testis that were localized to the Yq [54]. Since this discovery, the Y chromosome has been well annotated in different species and several functional genes identified [7]. Based on the expression pattern, these genes fall in two categories viz., the housekeeping genes which have X homologoues that escape X inactivation; the second group, consisting of the gene families expressed specifically in testes. The genes perform an array of regulatory functions and belong to diverse clasess such as histone lysine demethylases (*KDM5D* and *UTY*); the transcription factor (*ZFY*), spliceosomal component (*RBMY*); translation initiation factors (*DDX3Y* and *EIF1AY*); and the deubiquitinase (*USP9Y*) suggesting that the genes can govern expression of targets throughout the genome (Fig. 1).

## Genes and genetics of human Yq

Although the first demonstration of the functional capacity of the NRY of the human Y chromosome was in 1997, the involvement of this locus with male infertility was first made almost four decades ago. In 1976 Italian researchers, identified microscopically detectable deletions at the distal end of band q11 of the Y chromosome in six out of 1170 infertile males [55]. Analysis indicated that the fathers of two of the same six males had undeleted Y chromosomes, indicating that the deletions arose de novo and could be the underlying aetiology of their azoospermia. Based on these findings, the authors proposed the existence of a spermatogenesis factor, called the “azoospermia factor” [AZF] in the Yq locus. With the advent of the physical and molecular map of the human Y chromosome [56, 57] the studies in the AZF locus and male infertility were extensively propelled. Using a panel of molecular markers, a series of subfertile men who had deletions in the Yq were identified [58–60]. Based on the deletion analysis of these men three recurrently deleted non-overlapping sub-regions in proximal, middle, and distal Yq11 were defined and designated “AZFa,” “AZFb,” and “AZFc,” respectively [61]. While the *DAZ* (Deleted in Azoospermia) gene was considered as a strong candidate for male infertility [60] little was known about the existence of other genes within this locus. With the availability of the first complete sequence of AZFc locus [62] and later the detailed structure of the MSY including the AZFa, b and c regions [27] a large number of genes were identified.

Our understanding of the roles of these genes have mainly stemmed from the genetic analysis of oligozoospermic and azoospermic men where the presence and absence of these genes within the AZF a, b and c loci has been studied. Many of these studies are heterogeneous in nature and would have included men with compromised or absent spermatogenesis (mainly non obstructive oligozoospermia and its forms and azoospermia). For the sake of simplicity we have often used the term “infertile men” to describe these men which essentially mean that these studies would have been done in either azoospermic or oligozoospermic subjects or both.

## AZFa locus, its genes and its deletions

The AZFa encodes only single copy genes and is exclusively constituted by single-copy, ubiquitously expressed genes with X homologues that escape inactivation. Four genes have been mapped to AZFa.

## Ubiquitin specific peptidase 9, Y linked [*USP9Y*]

Earlier known as known as *DFFRY* or Drosophila fat facets related Y, *USP9Y* was the first gene identified in the AZFa sub-region with a length of 170 k base pairs [kb], consisting of 46 exons [63]. This gene encodes a large polypeptide of 2555 amino acids that is approximately 300 kilo Dalton [kDa] in size and belongs to the C19 cysteine peptidase family with protease activity specific to ubiquitin. *USP9Y* regulates the protein turnover by preventing degradation of proteins by the proteasome through the removal of ubiquitin from protein–ubiquitin conjugates [64] and also stabilizes the de-ubiquitinated target proteins, thus playing an important role in the development of germ cells in males [65]. *USP9Y* is ubiquitously expressed in adult and embryonic tissues and shares 91% identity with its X-homologue, *USP9X*, which escapes X-inactivation and is also expressed in many tissues. Deletion of Usp9x in the mouse causes sterility due to block in spermatogenesis during meiosis [66].

The *USPY* is one of the candidate genes of the AZFa as there exist infertile men with deletion or mutation in *USPY* [64, 67]. However, refuting this view, evidences are also available describing deletions of *USP9Y* in men with normal sperm count [63, 68] suggesting that *USP9Y* is not essential for normal sperm production and fertility in males.

Besides its role in spermatogenesis, studies have discovered that a nine-residue peptide derived from *USP9Y* represents a minor histocompatibility antigen [H-Y antigen] involved in graft rejection [69]. Recently, an association of gene fusion involving *USP9Y* (*TTTY15-USP9Y*) with prostate cancer has also been reported [70] suggesting its functions beyond regulation of sperm production.

## DEAD [asp-glu-ala-asp] box RNA helicases, Box 3, Y-linked [*DBY*]

*DBY*, also known as *DDX3Y*, was first identified by Lahn and Page [54] at the 5C region of the long arm of the Y chromosome at cytogenetic location Yq11.21 [53, 71]. *DBY*, which extends for 15.5 kb, consists of 17 exons and encodes a conserved ATP-dependent DEAD-box RNA helicase that is expressed only in germ cells with an alleged function at G1–S phase of the cell cycle [72]. *DBY* has a homologue on the X chromosome, *DBX* [53] and both of these genes have been reported to have > 95% sequence similarity while being expressed in two different stages of male germ cell line. While *DBY* protein expression is limited to only pre-meiotic male germ cells, the *DBX* protein is expressed in post-meiotic spermatids and in multiple somatic tissues [71].

Deletion analysis for the AZFa region in infertile males has revealed that males lacking *DBY* exhibit either Sertoli Cell Only syndrome [SCOS] or severe hypospermatogenesis suggesting that *DBY* plays a key role in the spermatogenic process [73, 74]. *DBY* protein has been reported to control the translation initiation of cyclin E1, which is essential for cell cycle progression from the G1 to S phase [75]. In drosophila, the *DBY* homologue, *Belle,* is essential for mitotic progression and survival of germline stem cells and spermatogonia [76]. Developmentally, *DBY* expression is initiated in the germ cells of human testis by 17 weeks of gestation implying that this protein may play a role in early spermatogonial proliferation [72]. This also suggests that, in males with AZFa deletion; germ cell depletion may begin prenatally. Indeed, complementation with *DBY* on the AZFa background, improved the formation of germ cell like cells from induced pluoripotent stem cells with AZFa deletion [77] suggesting that *DBY* functions in the earliest stages of human germ cell development.

## Ubiquitously transcribed tetratricopeptide repeat containing, Y linked [*UTY*]

*UTY* and Ubiquitously transcribed tetratricopeptidete repeat, X [*UTX*] genes are members of the tetratricopeptide repeat [TPR] protein family which occur in proteins that control mitosis. The *UTY* gene is located at the 5C band of AZFa contains 50 exons and a 3’UTR region with several polyadenylation signals [78]. Two *UTY* transcripts are detected in various human tissues such as spleen, thymus, prostate, testis, intestine, colon, and in cells such as leukocytes, but is not transcribed in ovary [53].

The *UTY* encodes a male-specific histone demethylase that catalyzes trimethylated ‘Lys-27’ [H3K27me3] demethylation in histone H3 of DNA. *UTY* is also involved in protein-protein interactions and may act as a chaperone [79]. Reports also suggest that the *UTY* protein is a minor histocompatibility antigen that may induce graft rejection of stem cell grafts of males [80]. A recent study [81] has suggested that the *UTY* is involved in a transcriptional regulatory network that is essential for prostate differentiation and that disruption of this network predisposes males to prostate cancer. Another study [82] reported the occurrence of *UTY* copy number variation in males afflicted with urothelial bladder cancer. The role of *UTY* in testis is not clear. Several missense mutations in *UTY* are reported in dbSNP database and computational analysis of some of these, have been shown to be deleterious [80]. However the fertility status of these men is unknown.

## Thymosin beta 4 Y linked, [*TB4Y*]

*TB4Y*, has been mapped to region 5D on the long arm of the human Y chromosome. *TB4Y* is expressed in various tissues, exists in a single copy and showing about 93% sequence similarity to its X chromosome homolog *TB4X* [53]. *TB4Y* encodes a novel human leukocyte antigen [HLA]-A*3303-restricted, minor histocompatibility antigen [83] and is a key activator of natural killer cell cytotoxicity [84]. The involvement of *TBY4* in testicular functions is unknown.

## AZFb locus, its genes and its deletions

**Fig. 2:**
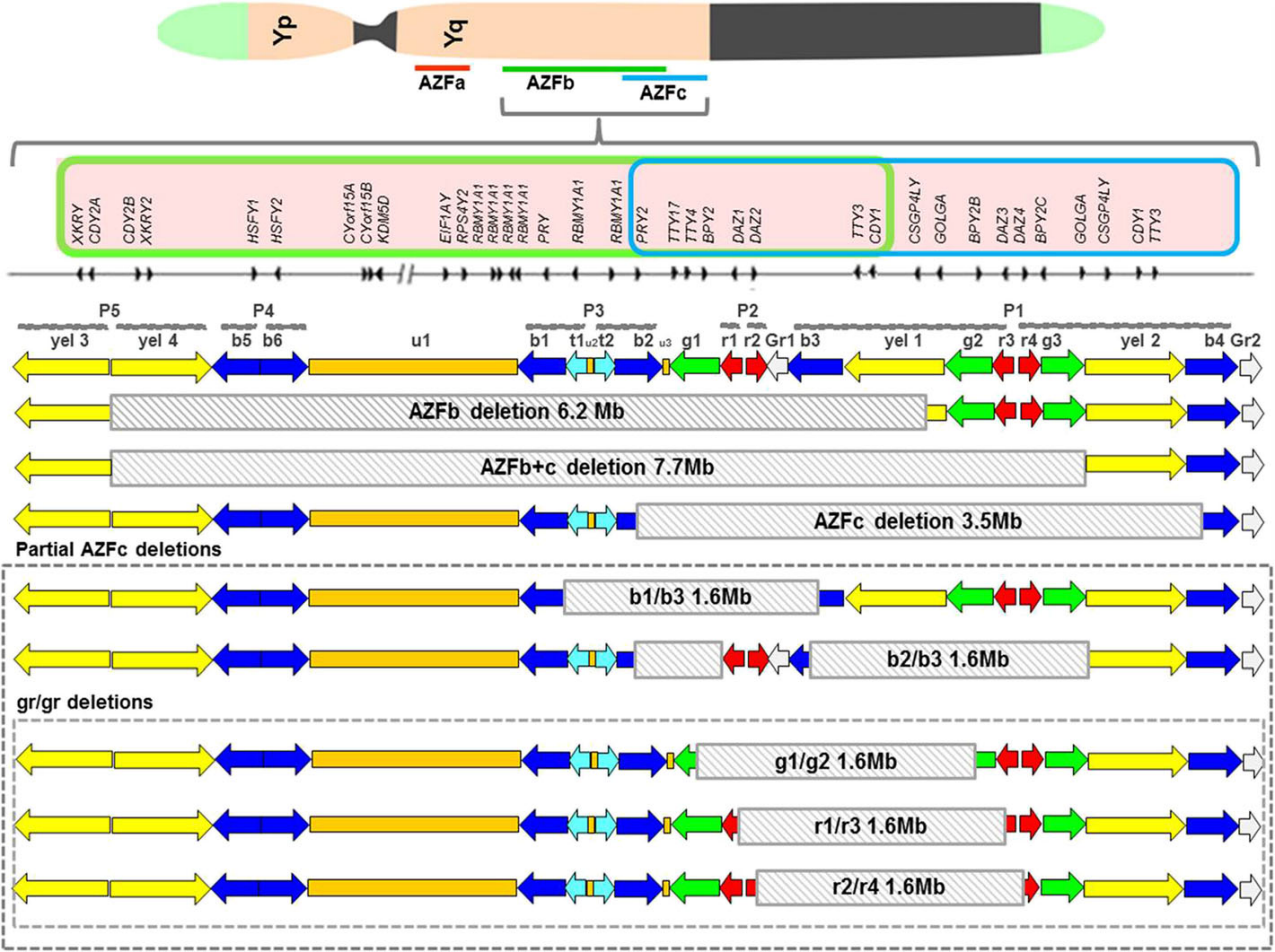
Schematic organization of the AZFb and c loci depicting how the various microdeletions arise. The AZFb and c regions are located in the euchromatic region on the Yq. Both regions share a number of genes [pink box], the genes present in the AZFb region are shown in the green box while the genes present in the AZFc region are present in the blue box. The grey arrows depict the orientation of the genes and the grey bars depict the organisation of the amplicons into palindromes [P1 to P5]. The AZFb and AZFc loci are composed of numerous stretches of ampliconic sequences [block arrows] which are annotated as six colour-coded sequence families (yellow, blue, turquoise, green, red and grey) called amplicons. The size and orientation of the coloured arrows represents the length and orientation of the arrows. AZFb is defined by the P5/proximal P1 deletion (yel3/yel1) which removes 6.23 Mb of DNA and AZFc by the b2/b4 deletion which removes 3.5 Mb of DNA. The partial AZFc deletions b1/b3, b2/b3 and the three variations of the gr/gr deletions [g1/g2], [r1/r3] and [r2/r4] [in dotted box] remove almost half of the AZFc gene content. The shaded block depicts the exact location of the deletion. The information of the map is adapted from published data ([6, 27], and [62])

The AZFb locus is located in the central region of Yq11 [intervals 5M-6B] and spans 3.2 Mb of which 1.5 Mb overlaps with AZFc (Figs. 1 and 2). The AZFb region has a complex genomic structure and contains three single-copy regions, a Y chromosome specific repeated DNA family [DYZ] 19 satellite repeat array and 14 multi-copy sequence units called amplicons (Fig. 2). These amplicons are organized into six sequence families, with intra-family homology levels > 99%. Amplicon families are defined by a specific colour code [yellow, blue, turquoise, green, red or grey] and each family member is identified by a numeral (Fig. 2). Of the 14 amplicon units, seven [yel3, yel4, b5, b6, b1, t1, t2] are restricted to AZFb, while the remaining are shared with AZFc. Amplicons are also categorized by a higher-order structural organization based on symmetrical arrays of contiguous repeat units called palindromes. AZFb contains palindromes P2 to P5 and the proximal part of P1 (Fig. 2). The presence of extensive ampliconic domains in AZFb allows for very complex rearrangements [62]. The critical AZFb interval necessary for spermatocyte maturation stretches from the center of palindrome P5 to the proximal edge of P3 within the *RBMY1* cluster, an interval of over 4 Mb containing 13 coding genes [85].

The AZFb is prone to NAHR [non-allelic homologous recombination] with AZFc, resulting in two frequent deletions of, 6.23 and 7.7 Mb. The 6.23 Mb, complete or classic AZFb deletion [P5/proximal P1] corresponds to the interval encompassed between amplicons yel3 and yel1 (Fig. 2) and occurs mainly due to unequal crossing over between homologous segments at their extremities [86]. This classical deletion overlaps within 1.5 Mb of the proximal portion of AZFc and leads to the loss of at least 32 coding genes and transcripts [87, 88]. Deletions of both AZFb and AZFc together occur in two breakpoints between P4/distal P1 (7.0 Mb, 38 gene copies removed) or between P5/distal P1 (7.7 Mb and 42 gene copies removed) [89]. The AZFb gene contains a total of five different single-copy transcription units as detailed in Fig. 2. However, not much is known about the biological functions of many of these genes.

## Chromosome Y open reading frame 15 [*CYorf15*]

*CYorf15A*and *CYorf15B* are single copy genes in AZFb which have an X homologue, *CXorf15*, thought to be involved with the taxilin family of proteins which are implicated in intracellular vesicle traffic [6].

## Ribosomal protein S4, Y linked [*RPS4Y2*]

Eukaryotic ribosomal protein S4 (S4e) is X-linked in mammals [90] and a Y linked homologue (*RPS4Y1*) is present in all primate lineages [91]. In humans, a second copy of the Y-linked gene (*RPS4Y2*) was described [27] which originated by duplication before the radiation of Old World Monkeys, approximately 35 million years ago [91]. The *RPS4Y1* is expressed in the testis and prostrate and is more highly expressed during spermatogenesis. It encodes a structurally conserved ribosomal protein subunit required for mRNA binding to the ribosome [92] and plays a role in the post-transcriptional regulation of the spermatogenic process.

## Eukaryotic Translation Initiation Factor 1A, Y linked [*EIF1AY*]

This gene is ubiquitously expressed and is a Y-linked member of the EIF-1A family—a family involved in translation initiation. The EIF-1A proteins enhance ribosome dissociation into subunits and stabilize the binding of the 43S complex to the end of capped RNA during protein biosynthesis. Studies implicate this gene with ischemic stroke [93, 94]. Widely expressed in multiple tissues, the biological roles of this gene are unknown. However, a close homologue of this gene in the mouse (*Eif2s3y*) along with SRY is sufficient to induce testicular differentiation and initiate spermatogenesis until the round spermatid stage in XX mice [95] suggesting its role in spermatogenesis.

## Lysine Demethylase 5D [KDM5D] / Selected Mouse cDNA, Y [*SMCY*]

This gene has several alternative names such as Jumonji At-Rich Interactive Domain 1D [*JARID1D*], Histocompatibility Y Antigen [*HY*] and H-Y Antigen [*HYA*]. It is thought that *KDM5D* plays a crucial role in chromosome condensation during meiosis by demethylating di- and tri-methylated H3K4 thus explaining the maturation arrest observed at the spermatocyte stage associated to AZFb deletions. The *KDM5D* enzyme is also known to form a protein complex with the MutS protein homolog 5 [MSH5] DNA repair factor during spermatogenesis which can be found on condensed DNA during the leptotene/zygotene stage, suggesting an involvement in male germ cell chromatin remodelling. Despite the apparently male germline-specific functions, this gene is ubiquitously expressed and is homologous to *KDM5C*, an X-borne gene. *KDM5D* has also been reported to have a tumour suppressor function in prostate cancer [96]. This gene regulates invasion-associated genes and the loss of *KDM5D* causes the cell to acquire invasiveness leading to the development of metastasis. A recent study [97] provides evidence that *KDM5D* plays an important role in determining docetaxel sensitivity, which is used in treating prostate cancer, by interacting with androgen receptor signalling and that its expression level is associated with clinical outcomes.

## X linked Kell Blood group precursor, Y linked [*XKRY*]

The *XKRY* exists in two copies in the yellow amplicon of AZFb (Fig. 2) that encodes a protein which is similar to XK, a putative membrane transport protein. By analysis of a panel of partial Y chromosomes this gene was mapped to region 5 L on the long arm of the human Y chromosome [53]. The functions of this protein is unknown, no role for it has been ascribed in spermatogenesis.

## Heat Shock transcription factor, Y linked [*HSFY*]

This gene maps to the blue amplicons with the two active copies located in b5 and b6 in the AZFb locus (Fig. 2). In the AZFb locus *HSFY* exists as two coding copies *HSFY1* and *HSFY2*. Although *HSFY* shows homology to the heat shock transcription factor-type (HSF) DNA-binding domain, it does not bind to heat shock elements and no HSFY-targeted promoters have been identified during spermatogenesis [98, 99]. *HSFY* has homologues at Xq28, *HSFX1* and *HSFX2* [27] but *HSFY* is expressed exclusively in the testis and principal cells of the epididymis.

That *HSFY* is functional and required for spermatogenesis is evident from the observations in four infertile males with a large 768 kb deletion around the P4 palindrome at the proximal end of the AZFb interval that resulted in loss of both *HSFY1* and *HSFY2*, and six non-coding transcription units *FAM41AY2*, *NCRNA00230B*, *TTTY9A*, *TTTY9B*, *NCRNA00185* and *TTTY14* [98]. Another study [99] also reported a partial AZFb deletion that removed the *HSFY* genes and three other genes [*KDM5D, CYorf15A and CYorf15B*] ostensibly only affecting the functional copies of *HSFY* in an azoospermic man.

The observations in genetic deletions are also backed by functional studies. *HSFY* is expressed in nuclei of germ cells with predominant expression in round spermatids. *HSFY* protein levels are decreased in testis of men with maturation arrest, associating this gene to the regulation of spermatogenesis [100–104].

## PTPN13-like Y linked, [*PTPBL*]-Related gene on Y [*PRY*]

**PRY** is a testis specific gene that encodes a protein similar to protein tyrosine phosphatase, non-receptor type 13 [105]. Two nearly identical copies of this gene, *PRY* and *PRY2*, map to the blue amplicons of the AZFb region (Fig. 2), with the two functional units being restricted to b1 and b2 [106].

The expression of *PRY* in germ cells is heterogeneous, with the protein being detected only in a few sperm and spermatids. Furthermore, *PRY* levels are increased in ejaculated sperm obtained from males with abnormal semen parameters, suggesting a link between its expression and defective spermatogenesis [107]. The *PRY* genes are thought to be involved in the regulation of apoptosis implicated in the removal of abnormal sperm [107]. Deletions that include the *PRY1* and *PRY2* genes have also been reported to cause meiotic arrest [108]. Studies reveal that in cases where all the genes in the AZFb region, excluding *RBMY* and *PRY* are deleted, there is hypospermatogenesis however if both *RBMY* and *PRY* are deleted, spermatogenesis is arrested completely [109]. This indicates that these two genes are the major genes involved in fertility [108, 110].

## Ribonucleic Acid [RNA]-Binding Motif, Y linked [*RBMY*]

The *RBMY* is one of the most important genes of the AZFb region with approximately six copies of this gene being dispersed within the Y chromosome [111]. The *RBMY1A1* gene family was identified [112] as a multicopy gene family designated *YRRM* (Y chromosome RNA recognition motif) and the first candidate azoospermia facto (Fig. 2). The proteins of the *RBM* gene family are characterised by the presence of an N-terminal RNA recognition motif [RRM] responsible for its interaction with target RNA molecules. Contrasting with the other RBM genes, *RBMY1A1* contains a C-terminal protein interaction repeat domain enriched in serine, arginine, glycine, and tyrosine [SRGY]. This serves as a probable regulatory region for the modulation of *RBMY1A1* function [113].

**Fig. 3:**
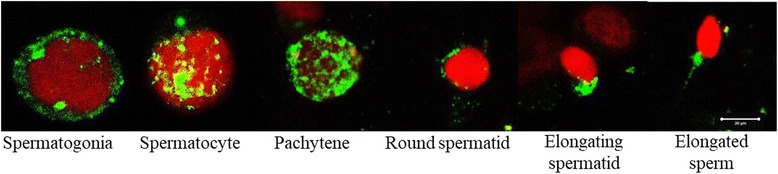
Expression of RBMY during human spermatogenesis. Human testicular cells were separated by mild collagenase digestion, smeared on slides and fixed in acetone. The cells were probed using an antibody against human RBMY (Santacruz Biotechnology Inc., sc – 14,572, USA) and detected using a FITC labeled secondary antibody. The cells were imaged under a fluorescent microscope and different stages were identified based on the cell and nuclear size. Green staining represents RBMY, red is nuclei. Bar represents 20 μm. For details of the methods see Abid et al. [115]

*RBMY1A1* is involved in several aspects of meiotic and pre-meiotic regulation via the establishment of multiple protein-protein and protein-RNA complexes. *RBMY1* encodes a testis-specific RNA binding protein that is expressed in the nuclei of spermatogonia, spermatocytes and round spermatids [114–118] and the expression of the protein is reduced in testis of men with AZFb deletions [117]. Interestingly, the subcellular distribution of *RBMY* differs in spermatogenic cells as they progress through meiosis [115], Fig. 3. In the spermatogonia, *RBMY* is localized as two foci, with one in the nucleolus and the other in the sub nuclear region. However in the spermatocytes, *RBMY* is distributed in a punctuate manner in the nucleus but the subnuclear foci is retained. In the pachytene cells, *RBMY* is spread along the length of the condensing chromosomes. Interestingly, in the round and elongating spermatids *RBMY* is excluded from the nucleus and restricted to the cytoplasm while it is retained in the mid-piece of ejaculated sperm (Fig. 3). These observations suggest that *RBMY* must have diverse functional roles during different stages of spermatogenesis. Indeed analysis of the human testicular *RBMY* bound transcriptome have led to identification of 20 target genes some of which are testis specific and have diverse cellular functions and is proposed to regulate alternate splicing during the course of spermatogenesis [119].

Molecular analysis of infertile men have has indicated a positive correlation between number of *RBMY1* copies and sperm count and motility [120]. Deletion of *RBMY1* copies leads to decrease in sperm count; however the persistence of the two proximal *RMBY1* copies is sufficient to avoid spermatogenic failure despite the total absence of the *PRY* gene [121]. These reports emphasized that the presence of *RMBY1* was sufficient for spermatogenesis to remain qualitatively complete although quantitatively reduced. Interestingly, unlike the humans, mice knockout of Rbmy undergo normal spermatogenesis but have abnormal spermatozoa [122] indicating that atleast in rodents Rbmy is dispensable for spermatogenesis but might be required in spermiogenesis. Indeed, even in humans, *RBMY* protein is detected in elongating spermatids and also in ejaculated sperm [115], Fig. 3. Sperm with high motility carry more *RBMY1* protein than those with relatively low motility [120]; inhibition of *RBMY* using a functionally neutralizing antibody inhibits sperm motility in vitro [115].

Beyond the testis, *RBMY* has been shown to play a role in liver cancers where *RBMY* was expressed exclusively in the testis of 36% of cases with hepatocellular carcinoma (HCCs), in 67% cases with hepatoblastoma and also in a liver cancer cell line [123, 124]. Gain of functions of *RBMY* in mouse fibroblast or in vivo in the liver results in vivo tumor formation [123, 124] downregulating *RBMY* in liver cancer cells reduces their tumorigenic potential [124]. *RBMY* is reportedly increased mainly in the hepatic stem cells of patients with HCC where it aberrantly signals GSK3β-WNT-β catenin signaling complex resulting in cell proliferation [124]. These observations imply that beyond the testis *RBMY* may act as an oncogene and this might explain the male predominance of various cancers including HCC.

## AZFc locus, its genes and its deletions

The AZFc is located at the distal part of deletion interval 6 (subintervals 6C–6E) on the Y chromosome (Figs. 1, 2). While the AZFa and AZFb regions are essential in initiating spermatogenesis, the AZFc region is essential to complete the process of spermatogenesis. The AZFc is the most commonly deleted region of the AZF locus in infertile men [27, 125].

The AZFc region spans 4.5 Mb and codes for 21 candidate genes and 11 families of transcription units (Fig. 2) that are exclusively expressed in the testis [100, 126]. This region contains six distinct families of amplicons; turquoise, grey and yellow, which occur twice in the AZFc locus, the green amplicon which occurs thrice and the blue and red amplicons which occur four times in the locus (Fig. 2). The members of each amplicon family are nearly identical and are arranged to form six large inverted repeats and three large direct repeats (Fig. 2). Three of the six inverted repeats are palindromes, or near palindromes, with large, inverted duplications containing much shorter intervening sequences. In the AZFc region, six distinct inverted repeat amplicon families are arranged in a complex repetitive pattern forming three palindromes that are believed to have formed as a result of the duplications and tandem inversions during evolution [62]. Together, palindromes P1, P2 and P3 encompass 4.0 of the 4.5 Mb of sequence (Fig. 2). Palindrome P1 is 1.5 Mb long with a span of 3 Mb and arm-to-arm identity of 99.97%. Within the arms of P1 lie two smaller palindromes, P1.1 and P1.2, each spanning 24 kb. The three uncoloured segments u1, u2 and u3 occur once each in this region (Fig. 2). u1 shows 70–85% identity to a locus on Yp, u2 shows 70–90% identity to an interspersed Y-specific repeated locus and u3 falls within a 65-kb block of 99.7% identity to Yp. The uncoloured 2 kb segments at the centres of the P1 and P2 palindromes are identical to each other (Fig. 2). The repetitive and palindromic nature of this locus and the fact that it is bordered with a highly repetitive heterochromatic region of Yq12 make this region highly susceptible to intra-chromosomal rearrangements during meiotic recombination thus making the AZFc locus prone to deletions, duplications and copy number variations of the eight gene families that are harboured within it [62].

There are no single-copy sequences in the AZFc (Fig. 2). The AZFc region includes 12 genes and transcription units, each present in a variable number of copies making a total of 32 copies. Amongst the various transcriptional units, only active copies of four protein-coding gene families map to the AZFc interval. These include the *PRY2, BPY2, DAZ* and *CDY1*. These genes locate to the blue, green, red and yellow-coded amplicons, respectively, with one transcription unit per amplicon copy (Fig. 2).

## Deleted in Azoospermia [*DAZ*]

*DAZ* originated on the Y chromosome as a transposition of a DNA segment containing the autosomal germ-cell specific gene *DAZL* almost 35 million years ago [87, 127]. Two autosomal *DAZ* homologs, *BOULE* and *DAZL*, have also been identified in humans with *BOULE* being considered a founding member of the *DAZ* gene family due to its conservation across the metazoan lineages [128, 129]. A loss of function mutation of the *Drosophila* homologue *boule* results in azoospermia thus emphasizing the role of *DAZ* in spermatogenesis [129].

*DAZ* was the first candidate gene to be isolated from the AZFc locus and was originally identified as a frequently deleted gene on the Y chromosome of infertile males [60, 130]. It was later found that the AZFc region contained palindromic duplications of *DAZ* as two clusters of four genes, *DAZ 1* and *2* and *DAZ 3* and *4* [131–133]. The four *DA*Z copies are expressed in spermatogonia, encoding an RNA-binding protein important for spermatogenesis [106] and these genes are expressed in all stages of germ cell development. Using human embryonic stem cells, it is shown that *DAZ* family genes function in germ cell formation and meiotic progression [134]. Therefore, all *DAZ* family genes are regarded critical for germ cell development.

Like the *RMBY* genes, the *DAZ* genes too, are known to contain an RNA recognition motif [RRM], expressed in the cytoplasm of pre-meiotic testicular germ cells, making these genes candidates for the maintenance of germ stem cell populations. The *DAZ* proteins are characterized by the presence of one or more *DAZ* repeat [24 amino acids rich in Asn, Tyr, and Gln residues], while in some cases, the *DAZ* motif is thought to mediate interaction with other proteins [135]. The human *DAZ* proteins carry out transportation, translational activation of developmentally regulated transcripts and their storage [136].

Deletion of *DAZ* accounts for 10% of cases of males with spermatogenic defects [136, 137]. Infertile males showing a loss of copies of *DAZ* genes are highly predisposed to azoospermia or severe oligozoospermia [138–140]. However due to the presence of the functional homologue (*DAZLA*) on human chromosome 3, a direct association between deletions in *DAZ* and azoospermia is difficult to conclude. Studies have also identified a single nucleotide polymorphism of *DAZL* that confers susceptibility to defects in spermatogenesis [141] and the deletion of the *DAZ1/DAZ2* doublet has been considered responsible for severe oligozoospermia, incomplete maturation arrest and for the testicular phenotype of residual spermiogenesis [140, 142].

While *DAZ* gene copies are deleted in men with complete and partial AZFc deletions, we and others have reported that individual *DAZ* copies can get deleted or duplicated even in absence of complete deletions [138, 140, 143–145]. While *DAZ* gene copy deletions/duplications are reported even in fertile men, deletion of *DAZ* and *CDY1* copies results in reduced sperm count and motility [140, 143]. These observations underscore the importance of *DAZ* genes in spermatogenesis. Functionally, the mechanism by which *DAZ* would regulate spermatogenesis remains unexplored, it is suspected to do so by regulation of RNA translation [146]. The targets of human *DAZ* gene have not been identified; human *DAZ* is known to interact with several other proteins that not necessarily have a role in protein translation [146] suggesting possibility of alternate mechanisms.

## Chromodomain Protein Y linked [*CDY*]

The human Y chromosome has two identical copies of this gene within the AZFc region [*CDY1A* and *CDY1B*] and a pair of closely related genes in the palindrome P5 [*CDY2A* and *CDY2B*, Fig. 2]. The *CDY1* gene encodes a protein containing an N-terminal chromatin-binding domain [chromo domain], which aids in regulation of gene expression, chromatin remodelling and encodes a histone acetyltransferase. This protein has been reported to concentrate in the round spermatid nucleus, where histone hyperacetylation occurs and causes the replacement of histones by the sperm-specific DNA packaging proteins, TNPs and PRMs [114, 147]. Although found only in primates, *CDY1* is believed to have been retroposed directly into MSY from a transcript of the autosomal gene *CDYL* more than 150 million years ago, making it one of the oldest genes on the Y chromosome [106].

The *CDY1* and *DAZ* families display autosomal homologues and hence some degree of functional redundancy between the Y-borne and the autosomal copies may partially account for the production of mature sperm in AZFc deleted males. One of the two copies of the *CDY1* gene is deleted in men with partial deletion of the AZFc locus [88, 105]. Based on analyses of gene copy deletions in the AZFc locus of infertile men, Machev et al. [148] discriminated four types of *DAZ*-*CDY1* partial deletions and found that only one deletion type, *DAZ3/4-CDY1a*, was associated with male infertility. In a similar study concomitant deletion of both *DAZ* and *CDY1* copies in males predisposes them to azoospermia or severe oligozoospermia [140, 149] deletion of *CDY1b* copy alone is also reported to be associated with oligo/azoospermia [150]. However, the fact that some men with *CDY1* deletions (alone or in combination with *DAZ*) are fertile/normozoopermic suggest that these genes are not indispensible for spermatogenesis [140, 151]. Beyond its function as a histone acetyltransferase, little is known about the molecular functions of *CDY1.*

## Basic Protein Y linked, 2 [*BPY2*]

The *BPY2* gene is expressed specifically in testis and its protein product is involved in male germ cell development. Three nearly identical copies of this gene exist on Y chromosome, *BPY2A*, *BPY2B* and *BPY2C*, of which two copies of are located at the boundaries of the gr/gr deletion, flanking the *DAZ* gene clusters (Fig. 2). *BPY2* is localized in the nuclei of spermatocytes, round spermatids and spermatogonia [152]. The *BPY2* gene encodes for a small positively charged protein which is thought to be involved in cytoskeletal regulation in spermatogenesis. Due to its small size and high charge, it is thought that *BPY* proteins may functionally interact with DNA in a manner that resembles chromatin-associated proteins such as histones and high mobility group proteins which are known to play a role in the regulation of processes such as transcription, replication, recombination and DNA repair [153]. The frequency of *BPY2* copy number alterations in infertile males is reported to be significantly high in the Chinese population [138] and also in Indian population (Modi et al. unpublished data). Some genetic variants in *BPY2* gene are associated with SCOs [154].

## Golgi autoantigen, golgi subfamily a, 2-like, Y-linked [*GOLGA2LY*]

*GOLGA* exists as two copies viz. *GOLGA2P2Y* and *GOLGA2P3Y* on the AZFc locus, arranged in opposite orientation in palindrome P1 (Fig. 2). This gene is reported to be transcribed and expressed only in the testis where it encodes a 108 amino acid protein [27, 106, 155]. However we have failed to amplify specific *GOLGA2LY* transcripts in the human testis, sequence analysis of some residual bands in RT-PCR experiments have been shown to arise out of non-specific amplifications from its homologs (Modi et al. unpublished data). *GOLGA2LY* protein has not been reported in any of the testicular proteomes [156]. Thus it is yet unclear if *GOLGA2LY* is a pseudogene or is transcriptionally active.

Partial AZFc deletion involves deletion of one of the two copies of the *GOLGA2LY* gene (Fig. 2). Although no function has been attributed to *GOLGA2LY*, it is interesting to note that males harbouring *GOLGA2P3Y* deletion have low sperm concentration and motility compared with males without deletion or with deletion of *GOLGA2P2Y*, suggesting the differential roles of the two copies spermatogenesis [138, 143]. The frequency of *GOLGA2P3Y* deletion is significantly higher in oligozoospermic men compared with normozoospermic men (odds ratio of 9), whereas the frequency of *GOLGA2P2Y* deletion was comparable between oligozoospermic and normozoospermic men [157]. Furthermore, men with *GOLGA2P3Y* deletion have reduced sperm concentration and motility compared with men without deletion or with deletion of *GOLGA2P2Y* [157] suggesting that loss of *GOLGA2P3Y* is an independent risk factor for oligozoospermia. However, assuming that the *GOLGA2LY* is a pseudogene and does not transcribe/translate in the testis, how these deletion result in infertility is difficult to ascertain. Positional shifts in the AZFc locus due to such deletions might offer a possible explanation.

## Chondroitin Sulfate Proteoglycan 4 Pseudogene 1, Y-linked [*CSPG4P1Y*]

Like the *GOLGA*, *CSPG4P1Y* the family of transcription units exist in two copies (Fig. 2) and is regarded as a pseudogene [32]. One of the two copies of the *CSPG4LY* gene is deleted in the b2/b3 deletion within the AZFc region [105]. Not much is known about the functions of this gene or its copies in maintenance of spermatogenesis.

## Testis-Specific Transcript, Y-Linked 4 [*TTY4*]

The *TTY4* gene has three copies (Fig. 2), *TTY4A*, *TTY4B* and *TTY4C*. This gene has not been studied in detail and is considered to be an RNA that does not encode any protein [88, 105]. *TTY4* copy deletions are rare in Indian population but, loss of one or more copies of *TTY4C* have been found to be associated with male infertility (Modi et al. unpublished data). In summary, it appears that the Y chromosome harbours genes that not only play integral roles in spermatogenesis but also in several aspects of human health. Several Y encoded genes are expressed not only in the testis but also in tissues involved in immune functions. [Refer Additional file 1: Table S2] However the putative functions of several Y borne genes still remains unknown and future studies may provide answers towards this end.

## Y chromosome microdeletions and male infertility

The human Y chromosome is genetically dynamic and is also prone to significant variation owing to the high proportion of segmental duplications which form the basis of the wide variety of deletions and duplications seen in various loci of this chromosome. Since the Yq locus contains large numbers of genes that are transcribed in the testis and have well defined role in spermatogenesis loss of these regions would cause infertility. Clinically, the Y chromosome alterations can be classified as (1) AZF deletions (complete loss of one or more of the AZF loci), (2) partial AZFc deletions and duplications and, (3) the gene copy number variations (CNVs). Described below are the prevalence and association of these deletions with male infertility.

## Yq microdeletions

Y chromosome microdeletions are small submicroscopic segmental deletions in the proximal Yq that remove the entire or parts of AZF region (complete deletions). Based on extensive deletion mapping studies in infertile men, five different deletions patterns on Yq have been reported [88]. However, in clinical practice, these are clubbed and termed as AZFa, AZFb, and AZFc deletions (Fig. 2). These deletions are one of the leading causes of spermatogenic failure and hence the screening for AZF deletions has become part of the routine diagnostic work-up of infertile men [158–160]. With the availability of the specific markers, simple PCR strategies to study the AZF locus are now available from various parts of the world that have shown the prevalence of Yq microdeletions. With a few exceptions of early reports, with refinements in the technologies, Yq microdeletions are detected exclusively in men with abnormal spermiograms and not been observed in large series of fertile men [158–160] suggesting that these deletions are the cause of failure of spermatogenesis and hence infertility. However, there are case reports where fathers of infertile men have been shown to carry these Yq deletions [161, 162] and this casts doubt on the whether these deletions themselves are sufficient to cause infertility or additional defects in the genome would be required to manifest the phenotype.

**Fig. 4:**
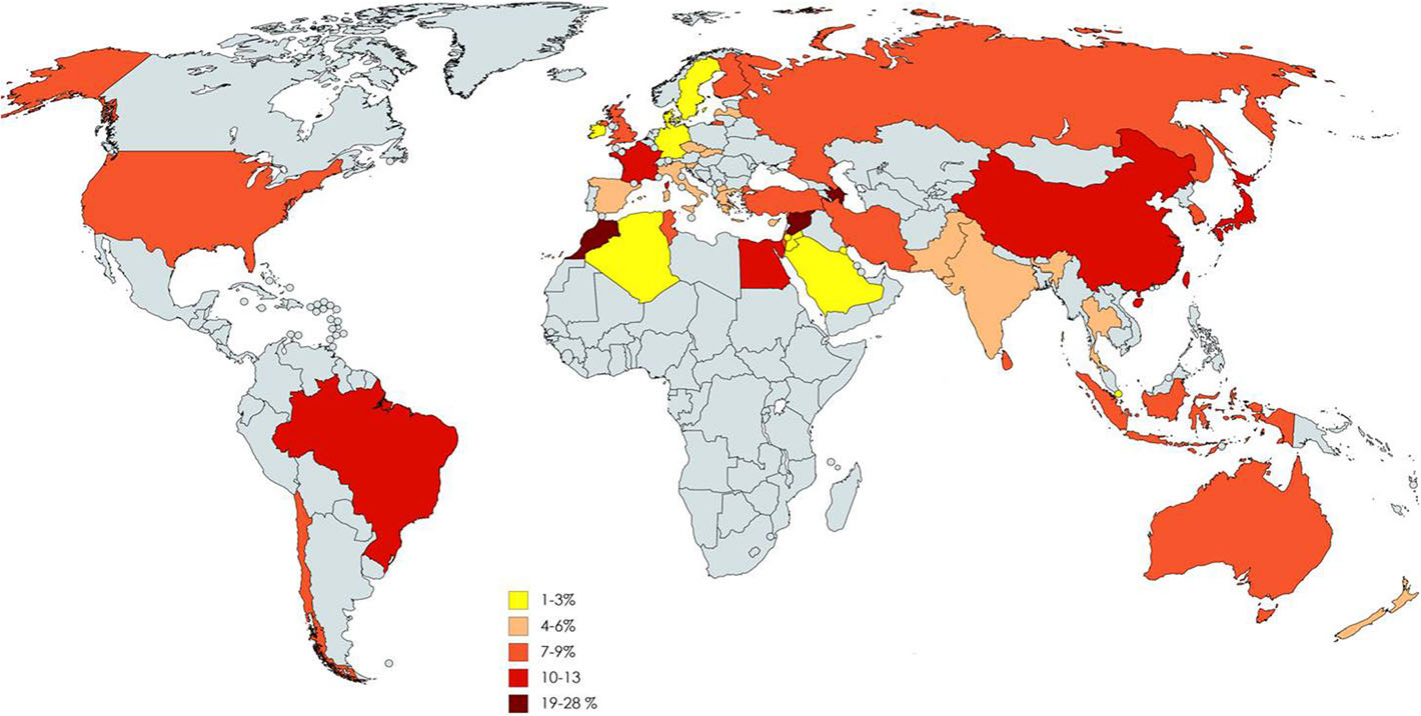
World map depicting the prevalence of Yq microdeletions in infertile males in different countries. The prevalence of Yq microdeletions in different countries of the world was estimated from published data of 40,127 Y chromosomes from infertile men. (oligozoospermic or azoospermic men). Only those articles published in English were considered and total number of infertile men studied and those having deletions were recorded along with the country. For each country data from different studies were pooled and the average estimated

**Fig. 5:**
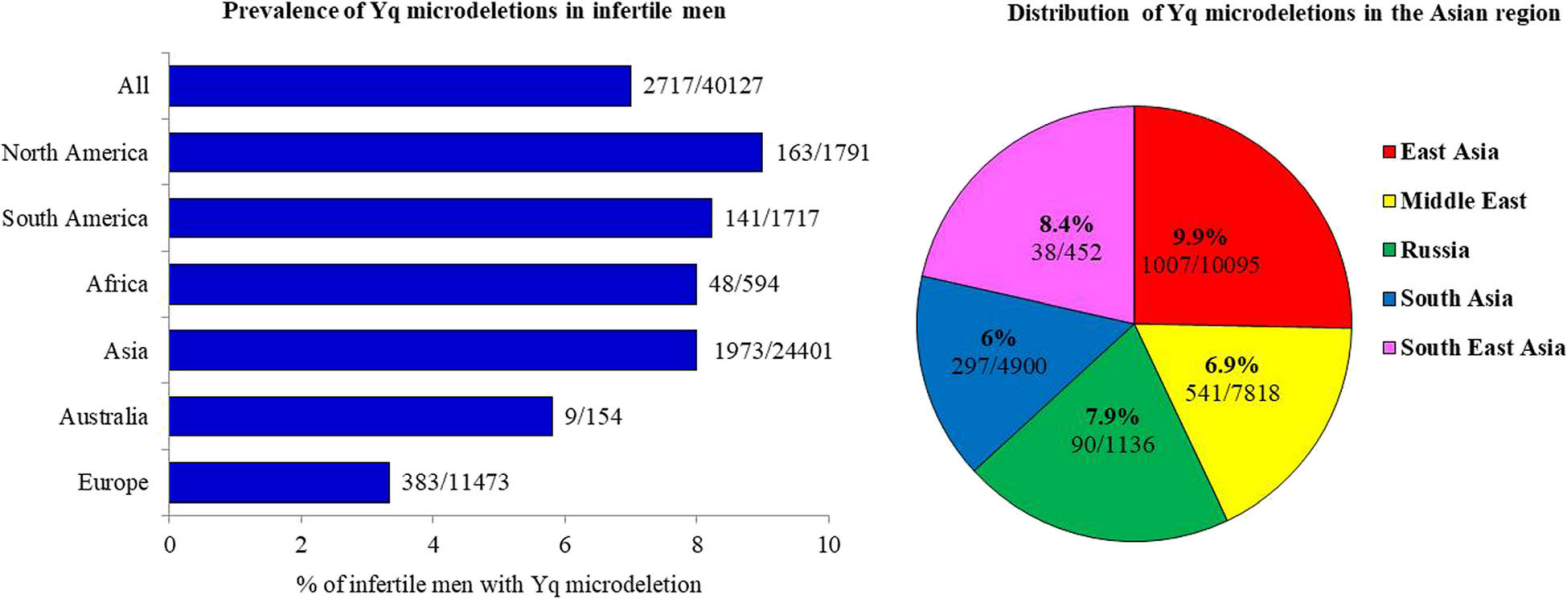
Prevalence of Yq microdeletions in infertile men. The average prevalence of the Yq microdeletions in different continents of the world was estimated from published data of 40,127 Y chromosomes from infertile men. Infertile men could be oligozoospermic or azoospermic men. Pie chart gives distribution of Yq microdeletions in the Asian region. The numbers were estimated from the data of Asian men based on geographical. In both the cases, only those articles published in English were considered and total number of infertile men studied and those having deletions were recorded along with the country. Data from different studies from same continent were pooled and the average estimated (for details see Additional file 1: Table S1)

Based on global data, Yq microdeletions are estimated to occur in about 1: 4000 men in the general population, but its frequency in infertile men is about 1:12. Of the > 30,000 Y chromosomes analysed for AZF microdeletions (by STS-PCR method), the global prevalence of AZF microdeletions in infertile men is estimated to be **7% (95% CL 6.74–6.79)**, (Fig. 4). As evident there is a wide variation in the frequency of Yq microdeletions in different parts of the world (Fig. 4), this could reflect underlying differences in sample size, methodology used and the population screened. The country wise published data of Yq deletion in infertile men is provided in Additional file 1: Table S1. However; pooled estimates based on geographic locations suggest that the lowest prevalence of Yq microdeletions is in Europe **(3% 95% CL 2.9–3.0)** and Australia **(5.3% 95% CL 5.9–7.8)**; while the rest of the world has an average of 8–9% (Fig. 5). What makes the Europeans and Australian infertile men less susceptible to Yq microdeletions warrants investigation. Amongst the Asians we observed that the highest prevalence of Yq microdeletions is amongst the East and South East Asians and lowest amongst South Asians (Fig. 5). This indicates that the susceptibility of the Yq to undergo microdeletions is perhaps race or ethnicity dependent. An influence of the Y chromosome genetic background (e.g. haplogroups) has been suggested to influence the susceptibility of partial AZFc deletions (see below). Whether these haplogroups also contribute to the occurrence of Yq microdeletions needs to be investigated. Since the global data is collated from diverse groups and not all studies have reported the ethnicity of the population investigated we are unable to determine the influence of different genetic background on prevalence of Yq microdeletions.

Irrespective of the population under study, the AZFc is most frequently deleted locus in infertile men [60–70%] followed by AZFa [0.5–4%], AZFb [1–5%] and AZFb+c [1–3%] deletion [11]. While these are global estimates, it is intriguing that the frequencies of the various AZF loci that are deleted in infertile men differ amongst various populations. While the frequency of AZFc deletion is lower in Indian population as compared to western counterparts (45% versus 60%), the frequency of AZFa deletion is almost double (11 versus 5%). Further, the frequency of double deletions (AZF a + b, b + c) is also higher in the Indian population as opposed to world literature [159]. A similar difference in the frequency of deletion of different AZF loci has been observed in Iranian population [163]. Also there are unusual combinations of deletions like the AZFa+c deletion observed in some populations but not the others [159, 164–166]. It is suggested that since the unusual combination of deletions are detected only by isolated markers and not confirmed by additional analyses these are perhaps methodological artefacts [158]. Nevertheless, it is important to note here that these deletions recurred in various populations and were detected using a variety of STS markers exclusively in infertile men; hence these deletion patterns should not be disregarded. Additional data will be needed to understand the molecular basis of such deletions.

## Phenotypic manifestations of Yq microdeletions

AZF deletions are specific to infertile men and hence it is appropriate to consider Y deletions as a cause of oligo/azoospermia rather than a cause of ‘infertility’. Overall, 25–55% of males with extreme testicular pathologies such as hypospermatogenesis, sperm maturation arrest and SCOS and 5–25% males with severe oligozoospermia or azoospermia harbour Y chromosome microdeletions [167]. However, depending on the AZF locus deleted the phenotypic manifestations reportedly vary.

### AZFa deletions

In general, deletions of the entire AZFa region invariably result in Sertoli cell only syndrome (SCOS) and azoospermia [58, 168–172]. Since genes within the AZFa locus are expressed in the germ cells prenatally, it is possible that the loss of these genes could lead to germ cell death developmentally leading to SCOS [72]. The partial AZFa deletions are however associated with phenotypes ranging from azoospermia to normozoospermia [173] indicating that the amount of genetic content lost is a critical determinant of azoospermia due to AZFa deletions. Hence the diagnosis of a complete deletion of the AZFa region implies the virtual impossibility to retrieve testicular spermatozoa for intracytoplasmic sperm injection (ICSI).

### AZFb deletions

The genes in the AZFb locus support the growth and maturity of sperm and are considered critical for efficient progression of sperm through meiosis into spermiogenesis. Not unexpectedly, patients with deletions of the AZFb region have a testicular phonotype of maturation arrest, frequently at the spermatocyte stage with an absence of post-meiotic germ cells in the majority of the tubules [6, 86, 174]. A clear correlation between the extent of the AZFb deletion and testicular histological findings is observed. Hypospermatogenesis is frequently observed among partial AZFb/b + c deleted specimens, but more severe findings are observed in complete AZFb and AZFb+c deleted specimens indicating that the AZFb deletions do not confer a severe phenotype but cause a block in spermatogenesis. The maturation arrest phenotype associated to AZFb deletions most probably stems from a combination of genetic disruption with structural defects in the chromosome [175].

It is generally accepted that the chance to retrieve mature sperm cells either from the ejaculate or via testicular sperm extraction is negligible in cases with AZFb deletion [175–177] although sperm have been retrieved from testis of men harbouring AZFb deletions [178–182]. While these differences could reflect the heterogeneity in extent of deletion in different patients; the likelihood of finding sperm cells in males with complete versus partial AZFb deletions is significantly lower [175].

### AZFc deletions

Men with AZFc deletions by far have the most variable phenotype ranging from complete azoospermia to mild oligozoospermia [126]. In general, the testis of men with AZFc deletions will have elongating spermatids and sperm can be retrieved in a reasonable numbers of AZFc deleted infertile males [166, 170]. In most men with AZFc deletion, spermatogenesis is completed, but on a reduced scale resulting in oligozoospermia [171].

By semen analysis, complete AZFc deletions are associated with drastic reduction in sperm count and most AZFc deleted males are severely oligozoospermic [158, 159, 171] however some may even be azoospermic [158, 159]. There also exist rare AZFc deleted males who have conceived multiple children naturally but all of the sons of these males have been found to be infertile [151, 161, 162].

While the AZFc deletions by themselves are less pathogenic, in prospective follow up case studies, it is observed that that in a subset of men with AZFc microdeletions there is progressive decline in sperm count and the patient progresses from oligozoospermia to its severe form or even become azoospermia [183–185]. This implies a temporally deteriorating effect of AZFc deletions on spermatogenesis leading to worsening in sperm numbers and quality. Thus although men with AZFc deletion may have sperm in the ejaculate, they must be offered semen cryopreservation to prevent invasive techniques like TESA in later stages of life for sperm retrieval.

## Partial AZFc deletions

The AZFc region is comprised of repeated sequences and palindromes hence making it most vulnerable to deletions. A complete deletion of AZFc involves the b2/b4 region which contains 12 genes and transcriptional units in multiple copy numbers (Fig. 2). In addition to b2/b4, the AZFc locus has many partial deletions that include b1/b3 (1.6 Mb), b2/b3 (1.8 Mb) and gr/gr (1.6 Mb) [186]. These deletions remove unique portions of the AZFc; however the genes removed are almost similar (Fig. 2). Three types of gr/gr deletions are reported that are caused by homologous recombination flanking the g1/g2, r1/r3 and r2/r4 amplicons in P1 and P2 palindromes in the AZFc (Fig. 2). However all the three are referred to as gr/gr and not classified further during analysis.

None of the partial deletions completely eliminate any gene family, but reduces the copy number of gene families with the exception of b1/b3 deletion that results in the loss of the six copies of *RBMY1* and both functional copies of PRY (Fig. 2). The gr/gr deletion results in loss of two of the four *DAZ* gene copies, one of the two *CDY1* and *BPY2* gene copies along with one of three copies of *BPY2* and two of the four *DAZ* genes. The b2/b3 deletion is derived from a gr/gr or b2/b3 inversion and removes almost the same gene content as gr/gr (Fig. 2).

## Prevalence of partial AZFc deletions and male infertility

The AZFc is of particular interest to both geneticist and a reproductive biologist as it harbours multicopy genes and provides an opportunity to study gene dosage effects in regulation of spermatogenesis. It is believed that the partial deletions lead to extensive copy number variations in the AZFc which might compromise the amounts of the protein produced thereby affecting spermatogenesis. This has formed the basis for analysis of the partial AZFc deletions in clinical evaluation of an infertile male. The ease of a well-designed PCR strategy has further fuelled this practice in various clinics and laboratories globally.

Since their first description, a wealth of information regarding partial AZFc deletions has accumulated in the past 15 years and it is clear that the association of partial AZFc deletions and male infertility is not as lucid as in the case of complete AZF deletions. This is because 1) there is wide heterogeneity in the types of partial AZFc deletions and 2) fertile and normozoospermic men harbour the AZFc partial deletions. Thus doubts have been cast on the involvement of AZFc partial deletions and male infertility. Three well designed meta-analysis and one study of > 20,000 Y chromosomes are published in recent times that have analysed the association of partial AZFc deletions and male infertility [126, 167, 187, 188]. Described below is the picture that has emerged out of these studies with large sample sizes.

**Fig. 6:**
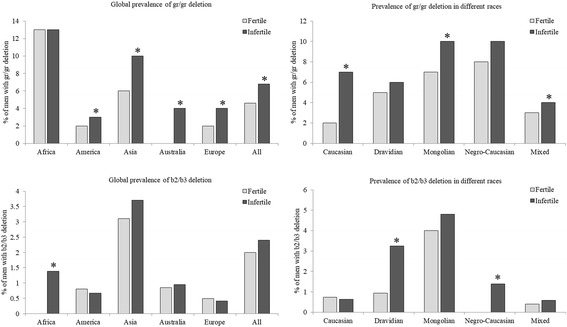
Association of gr/gr and b2/b3 deletions with male infertility. Data was obtained from previous studies [105, 126, 167, 188]. Data for gr/gr is derived out of 10,978 and 6704 Y chromosomes from infertile and fertile men respectively. For b2/b3 the data is derived out of 9981 and 5990 Y chromosomes from fertile and infertile men respectively. Infertile men could be oligozoospermic or azoospermic men. Fertile men would be normozoospermic/proven fertile men with unknown sperm counts. Data was divided based on continents or by race. * indicates value significantly different form fertile counterpart

Several controversies exist about the association of gr/gr deletion with male infertility. Early on it was reported that the gr/gr deletion was present in 3.8% of infertile males and 2.2% of fertile males (of unknown sperm counts) but absent in normozoospermic males [186] suggesting that the gr/gr deletion was associated with decreased sperm production resulting from the reduced dose of AZFc genes. However subsequent studies in many populations have yielded controversial findings where gr/gr deletions have been identified even in fertile males with normal sperm counts (see [167, 187]). While some studies have suggested that frequency of gr/gr deletion is significantly higher in infertile men as compared to fertile/normozoospermic controls; others have reported no such association. In two meta-analysis studies [167, 187] a significant association of gr/gr deletions and male infertility has however been observed. We analysed the available data [167] and observed that of the of the 10,978 Y chromosomes analysed from infertile men and 6704 from fertile/normozoospermic controls, twice the number of infertile men had gr/gr deletions as compared to controls (Fig. 6); with an OR of 1.8. Interestingly, the gr/gr deletions are common in African and Asian men (10–15%), they occur at a frequency of less than 5% in other population (Fig. 6). Furthermore, the association of gr/gr deletion with male infertility is observed in Australian, Asian and European and Australian men; the association is weak or negligible in American men and African men. The association of gr/gr deletion and male infertility is also ethnicity dependent [167, 187]. Amongst the different races, the association of gr/gr deletions and male infertility is strongest in Caucasian men and weaker in Mongolian men, but no association is observed in Dravidian and Nigro-Caucasian lineages (Fig. 6). Why the prevalence of gr/gr deletions and it association to male infertility differ with populations (geographically and ethnicity) is a matter of debate. It is suggested that such differences in susceptibility gr/gr deletions to male infertility is dependent on the Y chromosome background mainly the haplogroups [6]. A second school of thought is the high heterogeneity in the gene copy deletions in men with gr/gr deletions. It is shown that not all men with gr/gr deletions have identical amounts of DNA lost. Based on copy number estimates of *DAZ* and *CDY1* genes, it is observed that only those men with two *DAZ* and one *CDY1* copy deleted will have azoospermia or oligozoospermia, retention of any one of these genes will be almost always be associated with normozoospermia [139, 140, 143, 145, 149]. Along with *DAZ* and *CDY1*, studies have also identified *GOLGA2LY* and *BPY2* copy numbers have a protective effect on fertility of men with gr/gr deletions [138, 143, 157]. Thus it appears that the gene content and the gene dosage effect in the AZFc locus is a determinant of fertility in men harbouring gr/gr deletions.

Nevertheless, irrespective of the population, in individual studies as well as pooled estimates men with gr/gr deletions have significantly lower sperm counts and motility as compared to non-deleted controls [126, 167, 187] indicating that the gr/gr deletion might not be a direct cause of infertility, but does compromise process of spermatogenesis leading to lower sperm counts.

## b2/b3 deletion

As compared to the gr/gr, the b2/b3 is a slightly a larger deletion (1.6 Mb versus 1.8 Mb) and derived as an inversion (Fig. 2). Unlike the gr/gr, the correlation of b2/b3 partial deletions with spermatogenic failure is uncertain. Analysis of 17,784 Y chromosomes from infertile men and 11,684 Y chromosomes from fertile/normozoospermic men [188], showed a poor (but statistically significant) correlation of b2/b3 deletion and male infertility (Fig. 6). Like in case of gr/gr, the prevalence and association of male infertility and b2/b3 deletion differs geographically and is based on the ethnicity of the study population. Irrespective of the fertility status, the prevalence of b2/b3 deletion is highest amongst Asians (~ 3%) and lowest in Europeans (~ 0.5%) and in both these cases there is no significant difference in its frequency in fertile and infertile men; no association of b2/b3 deletions with male infertility has been noted in Australian men (Fig. 6). Intriguingly, while gr/gr deletions is not significantly associated with male infertility in African men, the prevalence of b2/b3 deletion is higher in infertile men of African origin as compared to its normozoospermic/fertile counter parts (1.17 vs. 0.25) and this difference is significant (Fig. 6). A similar association is observed in Chilean men where b2/b3 deletions are exclusively reported in infertile men. However, the b2/b3 deletion does not seem to be associated with male infertility in American men [126].

Like, the gr/gr, the association of b2/b3 deletions and male infertility also seems to be ethnicity dependent. The b2/b3 deletion is strongly association with male infertility in Dravidian and Negro-Caucasian men, weekly in Mongolian men but not in Caucasian men (Fig. 6). This pattern seems to be complementary to gr/gr deletions. The gr/gr but not b2/b3 deletion is strongly associated with male infertility in Caucasian men, the b2/b3 but not gr/gr is associated with male infertility in Dravidian and Negro-Caucasian men. In the Mongolian population both deletion carry a risk of infertility albeit weakly (Fig. 6). These observations are interesting and suggest that irrespective of the type of deletion and the population, the loss of gene copies in AZFc increases the susceptibility of an individual to reduction in sperm count. However, why one kind of deletion is pathogenic in one population but not the other needs further investigation.

## b1/b3 deletion

The b1/b3 deletion differs from the gr/gr and b2/b3 deletions because this deletion encompasses the part of AZFb and results in loss of the six copies of RBMY1 and both the copies of PRY (Fig. 6). Very few studies have reported association of b1/b3 deletion and male infertility. In an analysis of 20,000 Y chromosomes [126], the b1/b3 deletion is estimated to occur in one of every 994 men but this deletion increases the risk of spermatogenetic failure (OR 2.5). However, this deletion is very infrequent in most population and hence its association with male infertility is not known in most populations.

## Copy Number Variation in Y chromosome and male infertility

A Copy Number Variation [CNV] is defined as a DNA segment longer than 1 kb with a variable copy number of genes in comparison with a reference genome [189]. CNVs produce phenotype via diverse mechanisms, including gene dosage, presence of an interrupting gene, creating a fusion gene, unmasking of recessive coding region, mutation or other functional SNPs and position effect. All the different AZF microdeletions can be considered as large CNVs because they modify the copy number of genes on the Y chromosome. Further, the unstable AZFc region is predisposed to undergo several types of rearrangements resulting in changes in the cumulative copy number of the gene families contained within it. These complete or partial AZF deletions leading to CNVs of genes within it compromise spermatogenesis [138, 140, 143, 157, 190]. In addition to these AZF loci, CNVs across the entire length of the Y chromosome have been investigated in male infertility [125, 191–193]. In a CNV analysis of the MSY in healthy males, large duplications encompassing the *AMELY* and *TBL1Y* genes, partial deletions of the *TSPY* cluster, a variation in the number of *RBMY* genes and duplication of two segments overlapping the AZFa region have been reported [194] indicating that the Y chromosome is highly polymorphic in terms of its genetic content in general population. To understand the association of gene CNVs and male infertility, 68 well-defined histopathologically confirmed cases of males with idiopathic testicular maturation arrest were analysed by SNP arrays [103]. The results revealed PAR 1 and PAR 2 CNVs in 5 cases, PAR 3 CNVs in 19 cases and *TSPY2* gene gain in 16 cases. Men with normal spermatogenesis have not been investigated in this study; it is difficult to ascertain the significance of these CNVs. However, the discovery of PAR CNVs is biologically significant as X and Y chromosome pairing is made possible by the PARs and aberrations in chromosome structure at the PARs due to these CNVs would logically disrupt meiotic synapsis resulting in gametogenic failure and can be one of the aetiological factors underlying maturation arrest. In addition, the PAR regions are rich in genes that escape X inactivation to maintain dosage compensation (Fig. 1) it is plausible that these CNVs (deletions or duplications) of PARs may result in under- or over-expression of these genes resulting in a pathological phenotype. However, with the exception of genes in AZFc, no direct studies have been done comparing the CNVs with gene expression and spermatogenesis arrest.

## Clinical significance of genetic testing of Y chromosome and male infertility

Although there is universal agreement on the need for chromosome analysis by karyotyping in the workup of male infertility, there is still a lack of consensus regarding the clinical utility of testing for Y chromosome microdeletions in azoospermic and oligozoospermic males. While the American Society of Reproductive Medicine recommends the use of both, karyotyping and Y chromosome microdeletion studies, in males preparing to undergo Intracytoplasmic Sperm Injection [ICSI], the National Institute for Health and Care Excellence recommends only karyotyping for this group of patients [4]. In which patients molecular screening of the Y chromosome should be performed remains a dilemma. Based on data of several thousands of patients, Yq microdeletions are found in a high proportion of patients with azoospermia or severe oligozoospermia; the occurrence of deletion in infertile patients with sperm concentration > 5 × 10^6^/mL is usually less [195]. However there are population specific differences [159] and in this scenario country specific guidelines are necessary. While analysis of Yq microdeletions is not indicated in patients with chromosomal abnormalities, obstructive azoospermia or hypogonadotropic hypogonadism, there are a number of examples of deletion carriers among non-idiopathic infertile men including Klinefelter syndrome and varicocele [159]. Therefore, the presence of any diagnosis accompanied by azoo- or severe oligozoospermia should be an indication for AZF testing. We recommend Yq microdeletion testing in routine clinical practice for the following reasons

1. Identifying the cause of infertility: The Y chromosome contains several genes required for spermatogenesis and loss of one or more of such genes can cause impairment of this process. By investigating the presence of Y chromosome microdeletions it is possible to determine the underlying genetic aetiology of male factor infertility and implement appropriate screening strategies for abnormal phenotypes. Knowledge regarding these deletions will also help clinicians to provide more effective solutions to problems faced by infertile couples. For example, low sperm count and motility can be treated with hormones, anti-oxidants and lifestyle changes to improve the seminogram. However, these strategies of treatment will fail if the cause of infertility is genetic. Also AZF screening is important before varicocoelectomy because deletion carriers will most likely not benefit from the surgical procedure. Therefore, if the male partner is detected with a deletion the couple can directly be offered assisted reproductive techniques [ART] and not subjected to medical treatments to improve sperm count and motility.
2. Predicting the prognosis of infertile males: While there exist numerous cases reports of AZFc deleted men can father a child, it is clinically observed that individuals harbouring Y chromosome microdeletions have progressive decline in sperm counts and can progress to azoospermia over time (discussed above). Thus males with mild or moderate oligozoospermia and Yq microdeletions would require a multiple followed-up for their possible progression to azoospermia. Thus the knowledge of the Yq status would aid in counselling these men and provide them an option of sperm cryopreservation for biological parenthood in future.
3. Predicting outcome of testicular sperm aspiration [TESA]: Most males with Y chromosome microdeletions would be infertile and have absence of or very few sperms in ejaculate. To achieve pregnancy, sperm can be retrieved directly from the testes using techniques like testicular sperm extraction [TESE] or testicular sperm aspiration [TESA]. The occurrence and type of Yq microdeletion has been found to correlate with testicular phenotype and chance of sperm retrieval. Hence, screening for Y chromosome microdeletions can aid to predict the success of obtaining biological parenthood before undertaking invasive procedures.
4. Predicting the success of ART: Most men with Yq microdeletions would require ART for biological parenthood. Although yet controversial, Studies have reported slower fertilization rate, poor embryo quality, impaired blastocyst rate and lower overall success of ART in men with AZF deletions [140, 157, 166, 195]. Hence, Yq microdeletion screening would also help in counselling couples regarding the probability of success rates after taking up ART to aid the couple in a rational decision making.
5. Prevention of vertical transmission of the genetic defects: With the advent of ICSI, several live births are reported in couples where the male partner has a Yq microdeletion. However, in all these cases, the Yq microdeletion is passed down to the male offspring who are also infertile, thus resulting in a 100% transmission of the genetic defect and infertility from the fathers. In some instances the partial AZFc deletion in the father has resulted in full deletion in the offspring [196] which is a definite case of spermatogenic failure. Thus Y chromosome microdeletion testing is highly recommended for all infertile males who opt for ICSI so that the couple can make an informed choice of having biological parenthood at the risk of perpetuating infertility in the family.
6. Risk of testicular cancers: The Y chromosome has long been a suspect candidate for gonadal cancers. In individuals with gonadal dysgenesis that bear a full or even partial fragments of Y chromosome (even in a few of the cells) have a high risk of developing gonadal tumours specifically gonadoblastoma [197–200]. In addition the Yq microdeletions and/or its CNVs are suggested to be a risk factor for the development of testicular cancer [201]. While these are limited observational studies, results of large case control studies are yet awaited. It is becoming increasingly apparent that beyond infertility, the knowledge of Yq microdeletions should also aid in predicting occurrence of cancers in men.
7. Neuropsychiatric disorders and Yq microdeletions: While Yq microdeletions are strongly associated with infertility, reports are now emerging that atleast a subset of men with Y chromosome defects have higher prevalence of mental disorders. In a recent analysis of 42 Chilean patients diagnosed with Yq microdeletions a significantly higher proportion of abnormal height was seen amongst patients with terminal AZFb+c deletions compared with infertile cases without microdeletions. Intriguingly, 5/42 men (11%) had some forms of neuropsychiatric disorders including two with bipolar disorder and three with severe clinical depression. Clinical histories also documented language delay, attention-deficit hyperactivity disorder (ADHD) and emotional and behavioural problems including anxiety and social disabilities in this cohort [202]. However none of the infertile patients with non-terminal Yq microdeletions had any medical history of neuropsychiatric abnormalities [202]. While further long term clinical follow-up data of men with Yq microdeletions is required; these preliminary observations do indicate the occurrence of other health risks beyond infertility in such men and this warrants testing of Y chromosome genetics in a clinical situation.

## Conclusion and future directions

Male infertility is a complex multifactorial condition that presents with highly heterogeneous phenotypes. The Y chromosome plays a central role in regulation of spermatogenesis as it harbours Y-linked genes that are expressed in the testis and involved in various processes during spermatogenesis. The importance of these genes is evident from the observations that the removal of these genes causes distinct pathological testis phenotypes. After 20 years from the first molecular definition of the AZF, Yq deletion screening has now become a routine test for infertile males in many countries to identify the cause of male infertility. With clear-cut cause–effect relationship with severely impaired spermatogenesis, this test is now of help in even determining the success rates of sperm retrieval and prediction of success of assisted reproduction. Further as the deletion has 100% transmission rate to male offspring’s, the couple needs to be aware that the males in the future generation will also be infertile. Further as the gr/gr deletions are known to undergo expansion leading to full deletions [138, 196] the couple should be aware that besides the obligatory transmission of a genetic risk factor for impaired sperm production (gr/gr deletion) to the male offspring, there is a higher risk for the transmission of a complete AZFc deletion, which is a causative factor for spermatogenic failure.

Beyond these immediate applications, there are some clinically relevant issues associated with Yq deletions that need urgent attention. Presently, there is no long term follow-up data of men harbouring Yq microdeletions and there is an urgent need for data on the health status of children born from AZF deletion carriers. It is especially relevant in two conditions 1) Increased risk of testicular cancers 2) Possible occurrence of neurological dysfunctions. Due to the instable nature of the deleted Y chromosome, there is an increased risk of the expansion of deletions which might be a viewed as a “genomic instability”. Such progressive loss of genetic material during spermatogenesis might lead to sex chromosome mosaicism in germ cells which might predispose them to testicular/germ cell tumours. However, little data exist on the incidence of testicular tumours in men with Yq microdeletions especially in second generation males born to fathers carrying the deletion. Secondly, recent reports have demonstrated a significantly higher deletion load not only on the sex chromosome but also on autosomes of infertile men ([203, 204]) indicate more widespread effects of such deletions on genomic stability. Coupled with the fact that many Y linked genes are also expressed in multiple tissues; how genomic instability and disturbances in gene expression owing to Yq deletions affect general physiological functions has not been investigated the long term implications of such effects are obscure. With the recent data on higher prevalence of neurological problems in infertile men with Yq deletions it is imperative that we carry out detailed analysis of men with Yq deletions with an outlook beyond infertility. We hope that careful clinical observations coupled with detailed genetic information will provide important insights into these unanswered basic questions and give a different perspective to the field of androgenetics.

## Additional file

Additional file 1: **Table S1**. Table showing prevalence of Yq microdeletions in different countries. Data for different countries were collected form PubMed. Only those articles published in English were considered. The total number of infertile men studied and those having deletions were recorded along with the country. For each country data from different studies were pooled and the percentage estimated. Infertile men could be oligozoospermic or azoospermic men. **Table S2.** Table enlisting the Genes on the PAR1, PAR2 and NRY of the human Y chromosome. Their location, putative functions, cellular expression in testis, method of detection, expression in other tissues, role in spermatogenesis have been discussed. [Source https://www.proteinatlas.org/] [accessed on 31st January 2018]. (DOCX 48 kb)

## Abbreviations

ADHD: Attention-deficit hyperactivity disorder
Alu: *Arthrobacter luteus*
*AMELX*: Amelogenin, X-Linked
*AMELY*: Amelogenin, Y-Linked
AZF: Azoospermia factor
*BPY2*: Basic Charge, Y-Linked, 2
*CDY*: Chromodomain Protein, Y linked
CNV: Copy number variations
*CSPG4P1Y*: Chondroitin Sulfate Proteoglycan 4 Pseudogene 1, Y-Linked
*CXorf15*: Chromosome X Open Reading Frame 15
*CYorf15*: Chromosome Y Open Reading Frame 15
*DAZ*: Deleted in Azoospermia
*DAZL*: Deleted in Azoospermia like
dbSNP: The Single Nucleotide Polymorphism database
*DBY*: DEAD [asp-glu-ala-asp] box RNA helicases, Box 3, Y-linked
*DDX3Y*: DEAD-Box Helicase 3, Y-Linked
*DFFRY*: Drosophila fat facets related Y
DNA: Deoxyribonucleic acid
DSB: Double stranded break
DYZ1: DNA Y chromosome Z [number of repetitive segments at one site] 1
*EIF1AY*: Eukaryotic Translation Initiation Factor 1A, Y linked
*FAM41AY2*: Family With Sequence Similarity 41 Member A, Y-Linked 2
*GOLGA2P2Y*: Golgi autoantigen, golgi subfamily a, 2-like, Y-linked
GSK3β –WNT: Glycogen synthase kinase 3 – wingless integrated pathway
HCC: Hepatocellular carcinoma
HLA: Human leukocyte antigen
HSFY: Heat Shock transcription factor, Y linked
*HSPRY3*: *Homo Sapiens* Sprouty homolog 3
*HY*: Histocompatibility Y Antigen
*HYA*: Histocompatibility Y Antigen
ICSI: Intracytoplasmic sperm injection
*JARID1D*: Jumonji At-Rich Interactive Domain 1D
KDa: kiloDalton
*KDM5D*: Lysine Demethylase 5D
Mb: Mega base pairs
MSH5: MutS protein homolog 5
MSY: Male specific region
NAHR: Non-allelic homologous recombination
*NCRNA00185*: Non-protein coding RNA 185
*NCRNA00230B*: Non-Protein Coding RNA 230B
NOA: Non obstructive azoospermia
NRY: Non-recombining region
OA: Obstructive azoospermia
PAR 2: Pseudoautosomal regions 2
PAR: Pseudoautosomal regions
PAR1: Pseudoautosomal regions 1
*PCDH11Y*: Protocadherin 11, Y linked
PRM: Protamine
*PRY*: PTPN13-like Y linked, [PTPBL]-Related gene on Y
*RBMY*: Ribonucleic Acid -Binding Motif, Y linked
*RPS4Y1*: Ribosomal protein S4, Y linked
RRM: RNA recognition motif
SCOS: Sertoli Cell Only syndrome
*SHOX*: Stature homeobox
*SMCY*: Selected cDNA on Y, Mouse, homolog of
*SOX3*: *Sry Box 3*
SPO11α: Sporulation 11
*SRY*: Sex determining region on Y
*SYBL1*: *Synaptobrevin like 1*
*TB4Y*: Thymosin beta 4 Y linked
*TBL1Y*: Transducin Beta-like 1Y
*TBLR1*: Transducin Beta-like 1 K linked receptor
TESA: Testicular sperm aspiration
TESE: Testicular sperm extraction
TNP: Transition Nuclear Protein
TPR: Tetratricopeptide repeat
*TSPY*: Testis-specific protein Y-linked
*TTTY*: Testis-Specific Transcript, Y-Linked
*USP9Y*: Ubiquitin specific peptidase 9, Y linked
UTR: Untranslated region
*UTY*: Ubiquitously transcribed tetratricopeptide repeat containing, Y linked
WHO: World Health Organisation
*XKRY*: X linked Kell Blood group precursor, Y linked
*ZFY*: Zinc Finger protein, Y linked

## References

[1] WHO (2010). Laboratory manual for the examination and processing of human semen.

[2] Agarwal A, Mulgund A, Hamada A, Chyatte MR (2015). A unique view on male infertility around the globe. Reprod Biol Endocrinol.

[3] Hotaling J, Carrell DT (2014). Clinical genetic testing for male factor infertility: current applications and future directions. Andrology.

[4] Colaco S, Lakdawala A, Modi D (2017). Role of Y chromosome microdeletions in the clinical evaluation of infertile males. MGM J Med Sci.

[5] Hughes JF, Page DC (2015). The biology and evolution of mammalian Y chromosomes. Annu Rev Genet.

[6] Navarro-Costa P, Gonçalves J, Plancha CE (2010). The AZFc region of the Y chromosome: at the crossroads between genetic diversity and male infertility. Hum Reprod Update.

[7] Bellott DW, Hughes JF, Skaletsky H, Brown LG, Pyntikova T, Cho TJ, Koutseva N, Zaghlul S, Graves T, Rock S, Kremitzki C (2014). Mammalian Y chromosomes retain widely expressed dosage-sensitive regulators. Nature.

[8] Kauppi L, Barchi M, Baudat F, Romanienko PJ, Keeney S, Jasin M (2011). Distinct properties of the XY pseudoautosomal region crucial for male meiosis. Science.

[9] Page DC, Mosher R, Simpson EM, Fisher EM, Mardon G, Pollack J, McGillivray B, de la Chapelle A, Brown LG (1987). The sex-determining region of the human Y chromosome encodes a finger protein. Cell.

[10] Barchi M, Roig I, Di Giacomo M, de Rooij DG, Keeney S, Jasin M (2008). ATM promotes the obligate XY crossover and both crossover control and chromosome axis integrity on autosomes. PLoS Genet.

[11] Hinch AG, Altemose N, Noor N, Donnelly P, Myers SR (2014). Recombination in the human pseudoautosomal region PAR1. PLoS Genet.

[12] Mohandas TK, Speed RM, Passage MB, Yen PH, Chandley AC (1992). Role of the pseudoautosomal region in sex-chromosome pairing during male meiosis: meiotic studies in a man with a deletion of distal Xp. Am J Hum Genet.

[13] Gabriel-Robez O, Rumpler Y, Ratomponirina C, Petit C, Levilliers J, Croquette MF, Couturier J (1990). Deletion of the pseudoautosomal region and lack of sex-chromosome pairing at pachytene in two infertile men carrying an X; Y translocation. Cytogenet Cell Genet.

[14] Shi Q, Martin RH (2001). Aneuploidy in human spermatozoa: FISH analysis in men with constitutional chromosomal abnormalities, and in infertile men. Reproduction.

[15] Hassold TJ, Sherman SL, Pettay D, Page DC, Jacobs PA (1991). XY chromosome nondisjunction in man is associated with diminished recombination in the pseudoautosomal region. Am J Hum Genet.

[16] Ross MT, Grafham DV, Coffey AJ, Scherer S, McLay K (2005). The DNA sequence of the human X chromosome. Nature.

[17] Ellison JW, Wardak Z, Young MF, GehronRobey P, Laig-Webster M, Chiong W (1997). PHOG, a candidate gene for involvement in the short stature of turner syndrome. Hum Mol Genet.

[18] Rao E, Weiss B, Fukami M, Rump A, Niesler B, Mertz A, Muroya K, Binder G, Kirsch S, Winkelmann M, Nordsiek G, Heinrich U, Breuning MH, Ranke MB, Rosenthal A, Ogata T, Rappold GA (1997). Pseudoautosomal deletions encompassing a novel homeobox gene cause growth failure in idiopathic short stature and turner syndrome. Nat Genet.

[19] Lencz T, Morgan TV, Athanasiou M, Dain B, Reed CR (2007). Converging evidence for a pseudoautosomal cytokine receptor gene locus in schizophrenia. Mol Psychiatry.

[20] Flaquer A, Jamra RA, Etterer K, Dı’az GO, Rivas F (2010). A new susceptibility locus for bipolar affective disorder in PAR1 on Xp22.3/Yp11.3. Am J Med Genet.

[21] Mangs Helena A, Morris BJ (2007). The human pseudoautosomal region (PAR): origin, function and future. Curr Genomics.

[22] Charchar FJ, Svartman M, El-Mogharbel N, Ventura M, Kirby P, Matarazzo MR, Ciccodicola A, Rocchi M, D’Esposito M, Graves JA (2003). Complex events in the evolution of the human pseudoautosomal region 2 (PAR2). Genome Res.

[23] Lopes AM, Ross N, Close J, Dagnall A, Amorim A, Crow TJ (2006). Inactivation status of PCDH11X: sexual dimorphisms in gene expression levels in brain. Hum Genet.

[24] Veerappa AM, Padakannaya P, Ramachandra NB (2013). Copy number variation-based polymorphism in a new pseudoautosomal region 3 (PAR3) of a human X-chromosome-transposed region (XTR) in the Y chromosome. Funct Integr Genomics.

[25] Manz E, Alkan M, Bühler E, Schmidtke J (1992). Arrangement of DYZ1 and DYZ2 repeats on the human Y-chromosome: a case with presence of DYZ1 and absence of DYZ2. Mol Cell Probes.

[26] Cotter PD, Norton ME (2005). Y chromosome heterochromatin variation detected at prenatal diagnosis. Prenat Diagn.

[27] Skaletsky H, Kuroda-Kawaguchi T, Minx PJ, Cordum HS, Hillier L, Brown LG, Repping S, Pyntikova T, Ali J, Bieri T (2003). The male-specific region of the human Y chromosome is a mosaic of discrete sequence classes. Nature.

[28] Maan AA, Eales J, Akbarov A, Rowland J, Xu X, Jobling MA, Charchar FJ, Tomaszewski M (2017). The Y chromosome: a blueprint for men’s health?. Eur J Hum Genet.

[29] Meyfour A, Pooyan P, Pahlavan S, Rezaei-Tavirani M, Gourabi H, Baharvand H, Salekdeh GH. Chromosome-centric human proteome project allies with developmental biology: a case study of the role of Y chromosome genes in organ development. J Prot Res. 2017; 10.1021/acs.jproteome.7b00446. 10.1021/acs.jproteome.7b00446 28914051

[30] Rengaraj D, Kwon WS, Pang MG (2015). Bioinformatics annotation of human Y chromosome-encoded protein pathways and interactions. J Prot Res.

[31] Jacobs PA, Strongs JA (1959). A case of human intersexuality having a possible XXY sex determining mechanism. Nature.

[32] Ford CE, Jones KW, Polani PE, De Almeida JC, Briggs JH (1959). A sex-chromosome anomaly in a case of gonadal dysgenesis (Turner’s syndrome). Lancet.

[33] Koopman P, Sinclair A, Lovell-Badge R (2016). Of sex and determination: marking 25 years of Randy, the sex-reversed mouse. Development.

[34] Sinclair AH, Berta P, Palmer MS, Hawkins JR, Griffiths BL, Smith MJ, Foster JW, Frischauf AM, Lovell-Badge R, Goodfellow PN (1990). A gene from the human sex-determining region encodes a protein with homology to a conserved DNA-binding motif. Nature.

[35] She ZY, Yang WX (2017). Sry and SoxE genes: how they participate in mammalian sex determination and gonadal development?. Semin Cell Dev Biol.

[36] Quinn A, Koopman P (2012). The molecular genetics of sex determination and sex reversal in mammals. Semin Reprod Med.

[37] Modi D, Shah C, Sachdeva G, Gadkar S, Bhartiya D, Puri C (2005). Ontogeny and cellular localization of SRY transcripts in the human testes and its detection in spermatozoa. Reproduction.

[38] Decarpentrie F, Vernet N, Mahadevaiah SK, Longepied G, Streichemberger E, Aknin-Seifer I, Ojarikre OA, Burgoyne PS, Metzler-Guillemain C, Mitchell MJ (2012). Human and mouse ZFY genes produce a conserved testis-specific transcript encoding a zinc finger protein with a short acidic domain and modified transactivation potential. Hum Mol Genet.

[39] Yamauchi Y, Riel JM, Ruthig V, Ward MA (2015). Mouse Y-encoded transcription factor Zfy2 is essential for sperm formation and function in assisted fertilization. PLoS Genet.

[40] Vernet N, Mahadevaiah SK, Yamauchi Y, Decarpentrie F, Mitchell MJ (2014). Mouse Y-linked Zfy1 and Zfy2 are expressed during the male-specific interphase between meiosis I and meiosis II and promote the 2nd meiotic division. PLoS Genet.

[41] Vernet N, Mahadevaiah SK, Decarpentrie F, Longepied G, de Rooij DG (2016). Mouse Y-encoded transcription factor Zfy2 is essential for sperm head Remodelling and sperm tail development. PLoS One.

[42] Nakasuji T, Ogonuki N, Chiba T, Kato T, Shiozawa K, Yamatoya K, Tanaka H, Kondo T, Miyado K, Miyasaka N, Kubota T, Ogura A, Asahara H (2017). Complementary critical functions of Zfy1 and Zfy2 in mouse spermatogenesis and reproduction. PLoS Genet.

[43] Müller U, Kirkels VG, Scheres JM (1992). Absence of turner stigmata in a 46, XYp-female. Hum Genet.

[44] Jobling MA, Lo IC, Turner DJ, Bowden GR, Lee AC, Xue Y, Carvalho-Silva D, Hurles ME, Adams SM, Chang YM, Kraaijenbrink T (2006). Structural variation on the short arm of the human Y chromosome: recurrent multigene deletions encompassing Amelogenin Y. Hum Mol Genet.

[45] Meyfour A, Ansari H, Pahlavan S, Mirshahvaladi S, Rezaei-Tavirani M, Gourabi H, Baharvand H, Salekdeh GH. Y chromosome missing protein, TBL1Y, may play an important role in cardiac differentiation. J Proteome Res. 2017; 10.1021/acs.jproteome.7b00391. 10.1021/acs.jproteome.7b00391 28853286

[46] Russo P, Siani A, Miller MA, Karanam S, Esposito T, Gianfrancesco F, Barba G, Lauria F, Strazzullo P, Cappuccio FP (2008). Genetic variants of Y chromosome are associated with a protective lipid profile in black men. Arterioscler Thromb Vasc Biol.

[47] Johansson MM, Lundin E, Qian X, Mirzazadeh M, Halvardson J, Darj E, Feuk L, Nilsson M, Jazin E. Spatial sexual dimorphism of X and Y homolog gene expression in the human central nervous system during early male development. Biol Sex Differ. 2016; 10.1186/s13293-015-0056-4. 10.1186/s13293-015-0056-4 PMC4710049 26759715

[48] Priddle TH, Crow TJ (2013). The protocadherin 11X/Y (PCDH11X/Y) gene pair as determinant of cerebral asymmetry in modern Homo Sapiens. Ann N Y Acad Sci.

[49] Speevak MD, Farrell SA (2011). Non-syndromic language delay in a child with disruption in the Protocadherin11X/Y gene pair. Am J Med Genet B Neuropsychiatr Genet.

[50] Li Y, Zhang DJ, Qiu Y, Kido T, Lau YF (2017). The Y-located proto-oncogene TSPY exacerbates and its X-homologue TSPX inhibits transactivation functions of androgen receptor and its constitutively active variants. Hum Mol Genet.

[51] Delbridge ML, Longepied G, Depetris D, Mattei MG, Disteche CM, Graves JA, Mitchell MJ (2004). TSPY, the candidate gonadoblastoma gene on the human Y chromosome, has a widely expressed homologue on the X-implications for Y chromosome evolution. Chromosome Res.

[52] Krausz C, Giachini C, Forti G (2010). TSPY and male fertility. Genes.

[53] Yang X, Leng X, Tu W, Liu Y, Xu J, Pei X, Ma Y, Yang D, Yang Y. Spermatogenic phenotype of testis-specific protein, Y-encoded, 1 (TSPY1) dosage deficiency is independent of variations in TSPY-like 1 (TSPYL1) and TSPY-like 5 (TSPYL5): a case-control study in a Han Chinese population. Reprod Fertil Dev. 2017; 10.1071/RD17146. 10.1071/RD17146 28847364

[54] Lahn BT, Page DC (1997). Functional coherence of the human Y chromosome. Science.

[55] Tiepolo L, Zuffardi O. Localization of factors controlling spermatogenesis in the nonfluorescent portion of the human Y chromosome long arm. Hum Genet. 1976;34(2):119–24. 10.1007/BF00278879 1002136

[56] Vergnaud G, Page DC, Simmler MC, Brown L, Rouyer F, Noel B, Botstein D, De La Chapelle A, Weissenbach J (1986). A deletion map of the human Y chromosome based on DNA hybridization. Am J Hum Genet.

[57] Vollrath D, Foote S, Hilton A, Brown LG, Beer-Romero P, Bogan JS, Page DC (1992). The human Y chromosome: a 43-interval map based on naturally occurring deletions. Science.

[58] Vogt PH (1996). Human Y chromosome function in male germ cell development. Adv Dev Biol 1992.

[59] Reijo R, Alagappan RK, Page DC, Patrizio P (1996). Severe oligozoospermia resulting from deletions of azoospermia factor gene on Y chromosome. Lancet.

[60] Reijo R, Lee TY, Salo P, Alagappan R, Brown LG, Rosenberg M, Rozen S, Jaffe T, Straus D, Hovatta O, de la Chapelle A (1995). Diverse spermatogenic defects in humans caused by Y chromosome deletions encompassing a novel RNA–binding protein gene. Nat Genet.

[61] Vog PH, Edelmann A, Kirsch S, Henegariu O, Hirschmann P, Kiesewetter F, Köhn FM, Schill WB, Farah S, Ramos C, Hartmann M (1996). Human Y chromosome azoospermia factors (AZF) mapped to different subregions in Yq11. Hum Mol Genet.

[62] Kuroda-Kawaguchi T, Skaletsky H, Brown LG, Minx PJ, Cordum HS, Waterston RH, Wilson RK, Silber S, Oates R, Rozen S, Page DC (2001). The AZFc region of the Y chromosome features massive palindromes and uniform recurrent deletions in infertile men. Nat Genet.

[63] Luddi A, Margollicci M, Gambera L, Serafini F, Cioni M, De Leo V, Balestri P, Piomboni P (2009). Spermatogenesis in a man with complete deletion of USP9Y. N Engl J Med.

[64] Brown GM, Furlong RA, Sargent CA, Erickson RP, Longepied G, Mitchell M, Jones MH, Hargreave TB, Cooke HJ, Affara NA (1998). Characterisation of the coding sequence and fine mapping of the human DFFRY gene and comparative expression analysis and mapping to the Sxr-b interval of the mouse Y chromosome of the Dffry gene. Hum Mol Genet.

[65] Ho LK, Jee SG, Soo KI, Woong KS, Jae-Seung P, Ha CC, Kunsoo R (2003). Ubiquitin-specific protease activity of USP9Y, a male infertility gene on the Y chromosome. Reprod Fertil Dev.

[66] Kishi K, Uchida A, Takase HM, Suzuki H, Kurohmaru M, Tsunekawa N, Kanai-Azuma M, Wood SA, Kanai Y. Spermatogonial deubiquitinase USP9X is essential for proper spermatogenesis in mice. Reproduction. 2017; 10.1530/REP-17-0184. 10.1530/REP-17-0184 28559472

[67] Sun C, Skaletsky H, Birren B, Devon K, Tang Z, Silber S, Oates R, Page DC (1999). An azoospermic man with a de novo point mutation in the Y-chromosomal gene USP9Y. Nat Genet.

[68] Tyler-Smith C, Krausz C (2009). The will-o’-the-wisp of genetics—hunting for the azoospermia factor gene. N Engl J Med.

[69] Vogt MHJ, de Paus RA, Voogt PJ, Willemze R, Falkenburg JHF (2000). DFFRY codes for a new human male-specific minor transplantation antigen involved in bone marrow graft rejection. Blood.

[70] Zhu Y, Ren S, Jing T, Cai X, Liu Y, Wang F, Zhang W, Shi X, Chen R, Shen J, Lu J (2015). Clinical utility of a novel urine-based gene fusion TTTY15-USP9Y in predicting prostate biopsy outcome. Urol Oncol.

[71] Ditton HJ, Zimmer J, Kamp C, Rajpert-De Meyts E, Vogt PH (2004). The AZFa gene DBY (DDX3Y) is widely transcribed but the protein is limited to the male germ cells by translation control. Hum Mol Genet.

[72] Gueler B, Sonne SB, Zimmer J, Hilscher B, Hilscher W, Græm N, Rajpert-De Meyts E, Vogt PH (2012). AZFa protein DDX3Y is differentially expressed in human male germ cells during development and in testicular tumours: new evidence for phenotypic plasticity of germ cells. Hum Reprod.

[73] Foresta C, Ferlin A, Moro E (2000). Deletion and expression analysis of AZFa genes on the human Y chromosome revealed a major role for DBY in male infertility. Hum Mol Genet.

[74] Lardone MC, Parodi DA, Valdevenito R, Ebensperger M, Piottante A, Madariaga M, Smith R, Pommer R, Zambrano N, Castro A (2007). Quantification of DDX3Y, RBMY1, DAZ and TSPY mRNAs in testes of patients with severe impairment of spermatogenesis. Mol Hum Reprod.

[75] Lai MC, Chang WC, Shieh SY, Tarn WY (2010). DDX3 regulates cell growth through translational control of cyclin E1. Mol Cell Biol.

[76] Kotov AA, Olenkina OM, Godneeva BK, Adashev VE, Olenina LV (2017). Progress in understanding the molecular functions of DDX3Y (DBY) in male germ cell development and maintenance. Biosci Trends.

[77] Ramathal C, Angulo B, Sukhwani M, Cui J, Durruthy-Durruthy J, Fang F, Schanes P, Turek PJ, Orwig KE, Pera RR (2015). DDX3Y gene rescue of a Y chromosome AZFa deletion restores germ cell formation and transcriptional programs. Sci Rep.

[78] Laaser I, Theis FJ, de Angelis MH, Kolb H-J, Adamski J (2011). Huge splicing frequency in human Y chromosomal UTY gene. OMICS.

[79] Walport LJ, Hopkinson RJ, Vollmar M, Madden SK, Gileadi C, Oppermann U, Schofield CJ, Johansson C (2014). Human UTY(KDM6C) is a male-specific Nϵ-methyl lysyl demethylase. J Biol Chem.

[80] Nailwal M, Chauhan JB (2017). Computational analysis of high risk missense variant in human UTY gene: a candidate gene of AZFa sub-region. J Reprod Infertil.

[81] Dutta A, Le Magnen C, Mitrofanova A, Ouyang X, Califano A, Abate-Shen C (2016). Identification of an NKX3. 1-G9a-UTY transcriptional regulatory network that controls prostate differentiation. Science.

[82] Ahn J, Kim KH, Park S, Ahn YH, Kim HY, Yoon H, Lee JH, Bang D, Lee DH (2016). Target sequencing and CRISPR/Cas editing reveal simultaneous loss of UTX and UTY in urothelial bladder cancer. Oncotarget.

[83] Torikai H, Akatsuka Y, Miyazaki M, Warren EH, Oba T, Tsujimura K, Motoyoshi K, Morishima Y, Kodera Y, Kuzushima K, Takahashi T (2004). A novel HLA-A*3303-restricted minor histocompatibility antigen encoded by an unconventional open reading frame of human TMSB4Y gene. J Immunol.

[84] Lee HR, Yoon SY, Kang HB, Park S, Kim KE, Cho YH, Kim S, Kim CW, Cho BJ, Lee WJ, Bang SI, Park H, Cho D (2009). Thymosin beta 4 enhances NK cell cytotoxicity mediated by ICAM-1. Immunol Lett.

[85] Kichine E, Rozé V, Di Cristofaro J, Taulier D, Navarro A, Streichemberger E, Decarpentrie F, Metzler-Guillemain C, Lévy N, Chiaroni J, Paquis-Flucklinger V (2011). HSFY genes and the P4 palindrome in the AZFb interval of the human Y chromosome are not required for spermatocyte maturation. Hum Reprod.

[86] Soares AR, Costa P, Silva J, Sousa M, Barros A, Fernandes S (2012). AZFb microdeletions and oligozoospermia—which mechanisms?. Fertil Steril.

[87] Ravel C, Chantot-Bastaraud S, El Houate B, Rouba H, Legendre M, Lorenço D, Mandelbaum J, Siffroi JP, McElreavey K (2009). Y-chromosome AZFc structural architecture and relationship to male fertility. Fertil Steril.

[88] Repping S, Skaletsky H, Lange J, Silber S, van der Veen F, Oates RD, Page DC, Rozen S (2002). Recombination between palindromes P5 and P1 on the human Y chromosome causes massive deletions and spermatogenic failure. Am J Hum Genet.

[89] Zorrilla M, Yatsenko AN (2013). The genetics of infertility: current status of the field. Curr Genet Med Rep.

[90] Fisher E, Beer-Romero P, Brown L, Ridley A, McNeil J, Lawrence J, Willard H, Bieber F, Page DC (1990). Homologous ribosomal protein genes on the human X and Y chromosomes: escape from X inactivation and possible implications for turner syndrome. Cell.

[91] Andrés O, Kellermann T, López-Giráldez F, Rozas J, Domingo-Roura X, Bosch M (2008). RPS4Y gene family evolution in primates. BMC Evol Biol.

[92] Lopes AM, Miguel RN, Sargent CA, Ellis PJ, Amorim A, Affara NA (2010). The human RPS4 paralogue on Yq11. 223 encodes a structurally conserved ribosomal protein and is preferentially expressed during spermatogenesis. BMC Mol Biol.

[93] Tian Y, Stamova B, Jickling GC, Xu H, Liu D, Ander BP, Bushnell C, Zhan X, Turner RJ, Davis RR, Verro P (2012). Y chromosome gene expression in the blood of male patients with ischemic stroke compared with male controls. Gend Med.

[94] Yu A, Zhang J, Liu H, Liu B, Meng L (2016). Identification of nondiabetic heart failure-associated genes by bioinformatics approaches in patients with dilated ischemic cardiomyopathy. Exp Ther Med.

[95] Yamauchi Y, Riel JM, Stoytcheva Z, Ward MA. Two Y genes can replace the entire Y chromosome for assisted reproduction in the mouse. Science. 2013;3:1242544. 10.1126/science.1242544 PMC3880637 24263135

[96] Li N, Dhar SS, Chen TY, Kan PY, Wei Y, Kim JH, Chan CH, Lin HK, Hung MC, Lee MG (2016). JARID1D is a suppressor and prognostic marker of prostate cancer invasion and metastasis. Cancer Res.

[97] Komura K, Jeong SH, Hinohara K, Qu F, Wang X, Hiraki M, Azuma H, Lee GS, Kantoff PW, Sweeney CJ (2016). Resistance to docetaxel in prostate cancer is associated with androgen receptor activation and loss of KDM5D expression. Proc Natl Acad Sci U S A.

[98] Zhang W, Shao Y, Qin Y, Wu Y (2016). Expression pattern of HSFY in the mouse testis and epididymis with and without heat stress. Cell Tissue Res.

[99] Vinci G, Raicu F, Popa L, Popa O, Cocos R, McElreavey K (2005). A deletion of a novel heat shock gene on the Y chromosome associated with azoospermia. Mol Hum Reprod.

[100] Yu XW, Wei ZT, Jiang YT, Zhang SL (2015). Y chromosome azoospermia factor region microdeletions and transmission characteristics in azoospermic and severe oligozoospermic patients. Int J Clin Exp Med.

[101] Shinka T, Sato Y, Chen G, Naroda T, Kinoshita K, Unemi Y, Tsuji K, Toida K, Iwamoto T, Nakahori Y (2004). Molecular characterization of heat shock-like factor encoded on the human Y chromosome, and implications for male infertility. Biol Reprod.

[102] Stahl PJ, Mielnik AN, Barbieri CE, Schlegel PN, Paduch DA (2012). Deletion or underexpression of the Y-chromosome genes CDY2 and HSFY is associated with maturation arrest in American men with nonobstructive azoospermia. Asian J Androl.

[103] Halder A, Kumar P, Jain M, Iyer VK (2017). Copy number variations in testicular maturation arrest. Andrology.

[104] Tessari A, Salata E, Ferlin A, Bartoloni L, Slongo ML, Foresta C. Characterization of HSFY, a novel AZFb gene on the Y chromosome with a possible role in human spermatogenesis. Mol Hum Reprod. 2004;10(4):253—8. 10.1093/molehr/gah036 14985478

[105] Repping S, van Daalen SK, Korver CM, Brown LG, Marszalek JD, Gianotten J, Oates RD, Silber S, van der Veen F, Page DC, Rozen S (2004). A family of human Y chromosomes has dispersed throughout northern Eurasia despite a 1.8-Mb deletion in the azoospermia factor c region. Genomics.

[106] Alechine E, Corach D (2014). High-throughput screening for spermatogenesis candidate genes in the AZFc region of the Y chromosome by multiplex real time PCR followed by high resolution melting analysis. PLoS One.

[107] Stouffs K, Lissens W, Verheyen G, Van Landuyt L, Goossens A, Tournaye H, Van Steirteghem A, Liebaers I (2004). Expression pattern of the Y-linked PRY gene suggests a function in apoptosis but not in spermatogenesis. Mol Hum Reprod.

[108] Vogt PH (1998). Human chromosome deletions in Yq11, AZF candidate gene and male infertility: history and update. Mol Hum Reprod.

[109] Ferlin A, Raicu F, Gatta V, Zuccarello D, Palka G, Foresta C (2007). Male infertility: role of genetic background. Reprod BioMed Online.

[110] O’Flynn O’Brien KL, Varghese AC, Agarwal A (2010). The genetic causes of male infertility: a review. Fertil Steril.

[111] Tahmasbpour E, Balasubramanian D, Agarwal A (2014). A multi-faceted approach to understanding male infertility: gene mutations, molecular defects and assisted reproductive techniques (ART). J Assist Reprod Genet.

[112] Ma K, Inglis JD, Sharkey A, Bickmore WA, Hill RE, Prosser EJ, Speed RM, Thomson EJ, Jobling M, Taylor K (1993). A Y chromosome gene family with RNA-binding protein homology: candidates for the azoospermia factor AZF controlling human spermatogenesis. Cell.

[113] Elliott DJ (2004). The role of potential splicing factors including RBMY, RBMX, hnRNPG-T and STAR proteins in spermatogenesis. Int J Androl.

[114] Vogt PH (2005). AZF deletions and Y chromosomal haplogroups: history and update based on sequence. Hum Reprod Update.

[115] Abid S, Sagare-Patil V, Gokral J, Modi D (2013). Cellular ontogeny of RBMY during human spermatogenesis and its role in sperm motility. J Biosci.

[116] Elliott DJ, Oghene K, Makarov G, Makarova O, Hargreave TB, Chandley AC, Eperon IC, Cooke HJ (1998). Dynamic changes in the subnuclear organisation of pre-mRNA splicing proteins and RBM during human germ cell development. J Cell Sci.

[117] Elliott DJ, Millar MR, Oghene K, Ross A, Kiesewetter F, Pryor J, McIntyre M, Hargreave TB, Saunders PT, Vogt PH, Chandley AC (1997). Expression of RBM in the nuclei of human germ cells is dependent on a critical region of the Y chromosome long arm. Proc Natl Acad Sci U S A.

[118] Alikhani M, Tabar MS, Mirshahvaladi S, Kheimeh A, Gilani MA, Sabbaghian M (2013). Expression analysis of RNA-binding motif gene on Y chromosome (RBMY) protein isoforms in testis tissue and a testicular germ cell cancer-derived cell line (NT2). Iran Biomed J.

[119] Zeng M, Liang S, Zhao S, Liu Y, Sun H, Zhang S (2011). Identifying mRNAs bound by human RBMY protein in the testis. J Reprod Dev.

[120] Yan Y, Yang X, Liu Y, Shen Y, Tu W, Dong Q, Yang D, Ma Y, Yang Y (2017). Copy number variation of functional RBMY1 is associated with sperm motility: an azoospermia factor-linked candidate for asthenozoospermia. Hum Reprod.

[121] Plotton I, Ducros C, Pugeat M, Morel Y, Lejeune H (2010). Transmissible microdeletion of the Y-chromosome encompassing two DAZ copies, four RBMY1 copies, and both PRY copies. Fertil Steril.

[122] Mahadevaiah SK, Odorisio T, Elliott DJ, Rattigan Á, Szot M, Laval SH, Washburn LL, McCarrey JR, Cattanach BM, Lovell-Badge R, Burgoyne PS (1998). Mouse homologues of the human AZF candidate gene RBM are expressed in spermatogonia and spermatids, and map to a Y chromosome deletion interval associated with a high incidence of sperm abnormalities. Hum Mol Genet.

[123] Tsuei DJ, Hsu HC, Lee PH, Jeng YM, Pu YS, Chen CN, Lee YC, Chou WC, Chang CJ, Ni YH, Chang MH (2004). RBMY, a male germ cell-specific RNA-binding protein, activated in human liver cancers and transforms rodent fibroblasts. Oncogene.

[124] Tsuei DJ, Lee PH, Peng HY, Lu SL, Su DS, Jeng YM, Hsu HC, Hsu SH, Wu JF, Ni YH, Chang MH (2011). Male germ cell-specific RNA binding protein RBMY: a new oncogene explaining male predominance in liver cancer. PLoS One.

[125] Song SH, Chiba K, Ramasamy R, Lamb DJ (2016). Recent advances in the genetics of testicular failure. Asian J Androl.

[126] Rozen SG, Marszalek JD, Irenze K, Skaletsky H, Brown LG, Oates RD, Silber SJ, Ardlie K, Page DC (2012). AZFc deletions and spermatogenic failure: a population-based survey of 20,000 Y chromosomes. Am J Hum Genet.

[127] Seboun E, Barbaux S, Bourgeron T, Nishi S, Algonik A, Egashira M, Nikkawa N, Bishop C, Fellous M, McElreavey K, Kasahara M (1997). Gene sequence, localization, and evolutionary conservation of DAZLA, a candidate male sterility gene. Genomics.

[128] Xu EY, Moore FL, Pera RA (2001). A gene family required for human germ cell development evolved from an ancient meiotic gene conserved in metazoans. Proc Natl Acad Sci U S A.

[129] Eberhart CG, Maines JZ, Wasserman SA (1996). Meiotic cell cycle requirement for a fly homologue of human deleted in Azoospermia. Nature.

[130] Foresta C, Moro E, Garolla A, Onisto M, Ferlin A. Y chromosome microdeletions in cryptorchidism and idiopathic infertility. J Clin Endocrinol Metab. 1999;84:3660—5. 10.1210/jcem.84.10.6077 10523011

[131] Saxena R, de Vries JW, Repping S, Alagappan RK, Skaletsky H, Brown LG (2000). Four *DAZ* genes in two clusters found in the *AZFc* region of the human Y chromosome. Genomics.

[132] Vogt PH, Fernandes S (2003). Polymorphic DAZ gene family in polymorphic structure of *AZFc* locus: art work for functional for human spermatogenesis?. APMIS.

[133] Kim B, Lee Y, Kim Y, Lee KH, Chun S, Rhee K (2009). Polymorphic expression of DAZ proteins in the human testis. Hum Reprod.

[134] Kee K, Angeles VT, Flores M, Nguyen HN, Reijo RA (2009). Human DAZL, *DAZ* and *BOULE* genes modulate primordial germ-cell and haploid gamete formation. Nature.

[135] Fu XF, Cheng SF, Wang LQ, Yin S, De Felici M, Shen W (2015). DAZ family proteins, key players for germ cell development. Int J Biol Sci.

[136] Ferlin A, Moro E, Garolla A, Foresta C (1999). Human male infertility and Y chromosome deletions: role of the AZF-candidate genes DAZ, RBM and DFFRY. Hum Reprod.

[137] Kuo PL, Wang ST, Lin YM, Lin YH, Teng YN, Hsu CC (2004). Expression profiles of the DAZ gene family in human testis with and without spermatogenic failure. Fertil Steril.

[138] Lu C, Jiang J, Zhang R, Wang Y, Xu M, Qin Y, Lin Y, Guo X, Ni B, Zhao Y, Diao N (2014). Gene copy number alterations in the azoospermia-associated AZFc region and their effect on spermatogenic impairment. Mol Hum Reprod.

[139] Shahid M, Dhillon VS, Khalil HS, Sexana A, Husain SA (2011). Associations of Y-chromosome subdeletion gr/gr with the prevalence of Y-chromosome haplogroups in infertile patients. Eur J Hum Genet.

[140] Sen S, Ambulkar P, Hinduja I, Zaveri K, Gokral J, Pal A, Modi D (2015). Susceptibility of gr/gr rearrangements to azoospermia or oligozoospermia is dependent on DAZ and CDY1 gene copy deletions. J Assist Reprod Genet.

[141] Teng YN, Lin YM, Lin YH, Tsao SY, Hsu CC, Lin SJ, Tsai WC, Kuo PL (2002). Association of a single-nucleotide polymorphism of the deleted-in-azoospermia-like gene with susceptibility to spermatogenic failure. J Clin Endocrinol Metab.

[142] Fernandes S, Huellen K, Goncalves J, Dukal H, Zeisler J, Rajpert De Meyts E, Skakkebaek NE, Habermann B, Krause W, Sousa M, Barros A, Vogt PH (2002). High frequency of DAZ1/DAZ2 gene deletions in patients with severe oligozoospermia. Mol Hum Reprod.

[143] Noordam MJ, Westerveld GH, Hovingh SE, van Daalen SK, Korver CM, van der Veen F, van Pelt AM, Repping S (2011). Gene copy number reduction in the azoospermia factor c (AZFc) region and its effect on total motile sperm count. Hum Mol Genet.

[144] Mozdarani H, Ghoraeian P, Mozdarani S, Fallahi P, Mohseni-Meybodi A (2017). High frequency of de novo DAZ microdeletion in sperm nuclei of subfertile men: possible involvement of genome instability in idiopathic male infertility. Hum Fertil.

[145] Alimardanian L, Saliminejad K, Razi S, Ahani A (2016). Analysis of partial azoospermia factor c deletion and DAZ copy number in azoospermia and severe oligozoospermia. Andrologia.

[146] Van Gompel MJ, Xu EY (2011). The roles of the DAZ family in spermatogenesis: more than just translation?. Spermatogenesis.

[147] Lahn BT, Tang ZL, Zhou J, Barndt RJ, Parvinen M, Allis CD, Page DC (2002). Previously uncharacterized histone acetyltransferases implicated in mammalian spermatogenesis. Proc Natl Acad Sci U S A.

[148] Machev N, Saut N, Longepied G, Terriou P, Navarro A, Levy N, Guichaoua M, Metzler-Guillemain C, Collignon P, Frances AM, Belougne J (2004). Sequence family variant loss from the AZFc interval of the human Y chromosome, but not gene copy loss, is strongly associated with male infertility. J Med Genet.

[149] Ghorbel M, Baklouti-Gargouri S, Keskes R, Chakroun N, Sellami A, Fakhfakh F, Ammar-Keskes L (2016). Gr/gr-DAZ2-DAZ4-CDY1b deletion is a high-risk factor for male infertility in Tunisian population. Gene.

[150] Ghorbel M, Baklouti-Gargouri S, Keskes R, Chakroun N, Sellami A, Fakhfakh F, Ammar-Keskes L (2014). Deletion of CDY1b copy of Y chromosome CDY1 gene is a risk factor of male infertility in Tunisian men. Gene.

[151] Saut N, Terriou P, Navarro A, Lévy N, Mitchell MJ (2000). The human Y chromosome genes BPY2, CDY1 and DAZ are not essential for sustained fertility. Mol Hum Reprod.

[152] Tse JY, Wong EY, Cheung AN, O WS, Tam PC, Yeung WS (2003). Specific expression of VCY2 in human male germ cells and its involvement in the pathogenesis of male infertility. Biol Reprod.

[153] Singh I, Ozturk N, Cordero J, Mehta A, Hasan D, Cosentino C, Sebastian C, Krüger M, Looso M, Carraro G, Bellusci S (2015). High mobility group protein-mediated transcription requires DNA damage marker γ-H2AX. Cell Res.

[154] Choi J, Koh E, Suzuki H, Maeda Y, Yoshida A, Namiki M (2007). Alu sequence variants of the BPY2 gene in proven fertile and infertile men with Sertoli cell-only phenotype. Int J Urol.

[155] Jangravi Z, Alikhani M, Arefnezhad B, Sharifi Tabar M, Taleahmad S, Karamzadeh R, Jadaliha M, Mousavi SA, Ahmadi Rastegar D, Parsamatin P, Vakilian H (2012). A fresh look at the male-specific region of the human Y chromosome. J Prot Res.

[156] Ahmadi Rastegar D, Sharifi Tabar M, Alikhani M, Parsamatin P, Sahraneshin Samani F, Sabbaghian M, Sadighi Gilani MA, Mohammad Ahadi A, Mohseni Meybodi A, Piryaei A, Ansari-Pour N (2015). Isoform-level gene expression profiles of human Y chromosome azoospermia factor genes and their X chromosome paralogs in the testicular tissue of non-obstructive azoospermia patients. J Prot Res.

[157] Sen S, Agarwal R, Ambulkar P, Hinduja I, Zaveri K, Gokral J, Pal A, Modi D (2016). Deletion of GOLGA2P3Y but not GOLGA2P2Y is a risk factor for oligozoospermia. Reprod BioMed Online.

[158] Krausz C, Hoefsloot L, Simoni M, Tuttelmann F, European Academy of Andrology, European Molecular Genetics Quality Network (2014). EAA/EMQN best practice guidelines for molecular diagnosis of Y-chromosomal microdeletions: state-of-the-art 2013. Andrology.

[159] Sen S, Pasi AR, Dada R, Shamsi MB, Modi D (2013). Y chromosome microdeletions in infertile men: prevalence, phenotypes and screening markers for the Indian population. J Assist Reprod Genet.

[160] Simoni M, Bakker E, Krausz C (2004). EAA/EMQN best practice guidelines for molecular diagnosis of y-chromosomal microdeletions. State of the art 2004. Int J Androl.

[161] Chang PL, Sauer MV, Brown S (1999). Y chromosome microdeletion in a father and his four infertile sons. Hum Reprod.

[162] Gatta V, Stuppia L, Calabrese G, Morizio E, Guanciali-Franchi P, Palka G (2002). A new case of Yq microdeletion transmitted from a normal father to two infertile sons. J Med Genet.

[163] Yousefi-Razin E, Nasiri MJ, Omrani MD (2016). Frequency of Y chromosome microdeletions among Iranian infertile men with azoospermia and severe oligozoospermia: a meta-analysis. J Reprod Infertil.

[164] Foresta C, Moro E, Ferlin A (2001). Y chromosome microdeletions and alterations of spermatogenesis. Endocr Rev.

[165] Massart A, Lissens W, Tournaye H, Stouffs K (2012). Genetic causes of spermatogenic failure. Asian J Androl.

[166] Simoni M, Tuttelman F, Gromoll J, Nieschalg E (2008). Clinical consequences of microdeletions of the Y chromosome: the extended Munster experience. Reprod BioMed Online.

[167] Bansal SK, Jaiswal D, Gupta N, Singh K, Dada R, Sankhwar SN, Gupta G, Rajender S (2016). Gr/gr deletions on Y-chromosome correlate with male infertility: an original study, meta-analyses, and trial sequential analyses. Sci Rep.

[168] Kamp C, Huellen K, Fernandes S, Sousa M, Schlegel PN, Mielnik A, Kleiman S, Yavetz H, Krause W, Küpker W, Johannisson R (2001). High deletion frequency of the complete AZFa sequence in men with Sertoli-cell-only syndrome. Mol Hum Reprod.

[169] Krausz C, Quintana-Murci L, McElreavey K (2000). Prognostic value of Y deletion analysis: what is the clinical prognostic value of Y chromosome microdeletion analysis?. Hum Reprod.

[170] Hopps CV, Mielnik A, Goldstein M, Palermo GD, Rosenwaks Z, Schlegel PN (2003). Detection of sperm in men with Y chromosome microdeletions of the AZFa, AZFb and AZFc regions. Hum Reprod.

[171] Abid S, Maitra A, Meherji P, Patel Z, Kadam S, Shah J, Shah R, Kulkarni V, Baburao V, Gokral J (2008). Clinical and laboratory evaluation of idiopathic male infertility in a secondary referral center in India. J Clin Lab Anal.

[172] Kleiman SE, Almog R, Yogev L, Hauser R, Lehavi O (2012). Screening for partial AZFa microdeletions in the Y chromosome of infertile men: is it of clinical relevance?. Fertil Steril.

[173] Wei W, Fitzgerald T, Ayub Q, Massaia A, Smith BB, Dominiczak AA, Morris AA, Porteous DD, Hurles ME, Tyler-Smith C, Xue Y (2015). Copy number variation in the human Y chromosome in the UK population. Hum Genet.

[174] Costa P, Gonçalves R, Ferrás C, Fernandes S, Fernandes AT, Sousa M, Barros A (2008). Identification of new breakpoints in AZFb and AZFc. Mol Hum Reprod.

[175] Kleiman SE, Yogev L, Lehavi O, Hauser R, Botchan A, Paz G, Yavetz H, Gamzu R (2011). The likelihood of finding mature sperm cells in men with AZFb or AZFb-c deletions: six new cases and a review of the literature (1994–2010). Fertil Steril.

[176] Jungwirth A, Giwercman A, Tournaye H, Diemer T, Kopa Z, Dohle G, Krausz C, EAU Working Group on Male Infertility (2012). European Association of Urology guidelines on male infertility: the 2012 update. Eur Urol.

[177] Sadeghi-Nejad H, Farrokhi F (2007). Genetics of azoospermia: current knowledge, clinical implications, and future directions. Part II: Y chromosome microdeletions. Urol J.

[178] Liu XY, Wang RX, Fu Y, Luo LL, Guo W, Liu RZ (2017). Outcomes of intracytoplasmic sperm injection in oligozoospermic men with Y chromosome AZFb or AZFc microdeletions. Andrologia.

[179] Zhang YS, Li LL, Xue LT, Zhang H, Zhu YY, Liu RZ (2016). Complete AZFb deletion of Y chromosome in an infertile male with severe oligoasthenozoospermia: case report and literature review. Urology.

[180] Shi YC, Cui YX, Zhou YC, Wei L, Jiang HT, Xia XY, Lu HY, Wang HY, Shang XJ, Zhu WM, Li XJ, Huang YF (2011). A rare Y chromosome constitutional rearrangement: a partial AZFb deletion and duplication within chromosome Yp in an infertile man with severe oligoasthenoteratozoospermia. Int J Androl.

[181] Stouffs K, Vloeberghs V, Gheldof A, Tournaye H, Seneca S (2017). Are AZFb deletions always incompatible with sperm production?. Andrology.

[182] Choi JM, Chung P, Veeck L, Mielnik A, Palermo GD, Schlegel PN (2004). AZF microdeletions of the Y chromosome and in vitro fertilization outcome. Fertil Steril.

[183] Simoni M, Gromoll J, Dworniczak B, Rolf C, Abshagen K, Kamischke A (1997). Screening for deletions of the Y chromosome involving the DAZ (deleted in Azoospermia) gene in azoospermia and severe oligozoospermia. Fertil Steril.

[184] Krausz C, Forti G (2006). Sperm cryopreservation in male infertility due to genetic disorders. Cell Tissue Bank.

[185] Fu L, Xiong DK, Ding XP, Li C, Zhang LY (2012). Genetic screening for chromosomal abnormalities and Y chromosome microdeletions in Chinese infertile men. J Assist Reprod Genet.

[186] Repping S, Skaletsky H, Brown L, van Daalen SK, Korver CM, Pyntikova T, Kuroda-Kawaguchi T, de Vries JW, Oates RD, Silber S, van der Veen F, Page DC, Rozen S (2003). Polymorphism for a 1.6-Mb deletion of the human Y chromosome persists through balance between recurrent mutation and haploid selection. Nat Genet.

[187] Stouffs K, Lissens W, Tournaye H, Haentjens P (2011). What about gr/gr deletions and male infertility? Systematic review and meta-analysis. Hum Reprod Update.

[188] Bansal SK, Gupta G, Rajender S (2016). Y chromosome b2/b3 deletions and male infertility: a comprehensive meta-analysis, trial sequential analysis and systematic review. Mutat Res Rev Mutat Res.

[189] Krausz C, Chianese C, Giachini C, Guarducci E, Laface I, Forti G (2011). The Y chromosome-linked copy number variations and male fertility. J Endocrinol Investig.

[190] Vodicka R, Vrtel R, Dusek L, Singh AR, Krizova K, Svacinova V, Horinova V, Dostal J, Oborna I, Brezinova J, Sobek A (2007). TSPY gene copy number as a potential new risk factor for male infertility. Reprod BioMed Online.

[191] Lee C, Iafrate AJ, Brothman AR (2007). Copy number variations and clinical cytogenetic diagnosis of constitutional disorders. Nat Genet.

[192] Tüttelmann F, Simoni M, Kliesch S, Ledig S, Dworniczak B, Wieacker P, Röpke A (2011). Copy number variants in patients with severe oligozoospermia and Sertoli-cell-only syndrome. PLoS One.

[193] Dong Y, Pan Y, Wang R, Zhang Z, Xi Q, Liu RZ (2015). Copy number variations in spermatogenic failure patients with chromosomal abnormalities and unexplained azoospermia. Genet Mol Res.

[194] Krausz C, Casamonti E (2017). Spermatogenic failure and the Y chromosome. Hum Genet.

[195] Mateu E, Rodrigo L, Martínez MC, Peinado V, Milán M, Gil-Salom M, Martínez-Jabaloyas JM, Remohí J, Pellicer A, Rubio C (2010). Aneuploidies in embryos and spermatozoa from patients with Y chromosome microdeletions. Fertil Steril.

[196] Zhang F, Lu C, Li Z, Xie P, Xia Y, Zhu X, Wu B, Cai X, Wang X, Qian J, Wang X (2007). Partial deletions are associated with an increased risk of complete deletion in AZFc: a new insight into the role of partial AZFc deletions in male infertility. J Med Genet.

[197] Modi D, Bhartiya D (2007). Y chromosome mosaicism and occurrence of gonadoblastoma in cases of turner syndrome and amenorrhoea. Reprod BioMed Online.

[198] Jørgensen A, Johansen ML, Juul A, Skakkebaek NE, Main KM, Rajpert-De Meyts E (2015). Pathogenesis of germ cell neoplasia in testicular dysgenesis and disorders of sex development. Semin Cell Dev Biol.

[199] Kido T, Lau YF (2015). Roles of the Y chromosome genes in human cancers. Asian J Androl.

[200] Huang H, Wang C, Tian Q (2017). Gonadal tumour risk in 292 phenotypic female patients with disorders of sex development containing Y chromosome or Y-derived sequence. Clin Endocrinol.

[201] Nathanson KL, Kanetsky PA, Hawes R, Vaughn DJ, Letrero R, Tucker K, Friedlander M, Phillips KA, Hogg D, Jewett MA, Lohynska R (2005). The Y deletion gr/gr and susceptibility to testicular germ cell tumor. Am J Hum Genet.

[202] Castro A, Rodríguez F, Flórez M, López P, Curotto B, Martínez D, Maturana A, Lardone MC, Palma C, Mericq V, Ebensperger M (2017). Pseudoautosomal abnormalities in terminal AZFb+ c deletions are associated with isochromosomes Yp and may lead to abnormal growth and neuropsychiatric function. Hum Reprod.

[203] Krausz C, Escamilla AR, Chianese C (2015). Genetics of male infertility: from research to clinic. Reproduction.

[204] Lopes AM, Aston KI, Thompson E, Carvalho F, Gonçalves J, Huang N, Matthiesen R, Noordam MJ, Quintela I, Ramu A, Seabra C (2013). Human spermatogenic failure purges deleterious mutation load from the autosomes and both sex chromosomes, including the gene DMRT1. PLoS Genet.

