# Supplementary material for: Genetics of the human Y chromosome and its association with male infertility
Source: Reprod Biol Endocrinol. 2018 Feb 17;16:14. doi: 10.1186/s12958-018-0330-5 (PMC5816366; doi:10.1186/s12958-018-0330-5)
Supplement: Supplementary file 1 — Table S1. Table showing prevalence of Yq microdeletions in different countries. Data for different countries were collected form PubMed. Only those articles published in English were considered. The total number of infertile men studied and those having deletions were recorded along with the country. For each country data from different studies were pooled and the percentage estimated. Infertile men could be oligozoospermic or azoospermic men. Table S2. Table enlisting the Genes on the PAR1, PAR2 and NRY of the human Y chromosome. Their location, putative functions, cellular expression in testis, method of detection, expression in other tissues, role in spermatogenesis have been discussed. [Source https://www.proteinatlas.org/] [accessed on 31st January 2018]. (DOCX 48 kb) [file 12958_2018_330_MOESM1_ESM.docx]

**Additional file 1**

| **Country** | **Total studied** | **Deleted** | **Percentage** |
| --- | --- | --- | --- |
| Algeria | 80 | 1 | 1 |
| America | 1791 | 163 | 9 |
| Argentina | 230 | 3 | 1 |
| Australia | 29 | 2 | 7 |
| Austria | 187 | 0 | 0 |
| Azerbaijan | 99 | 24 | 24 |
| Brazil | 474 | 50 | 11 |
| Chile | 1010 | 88 | 9 |
| China | 6979 | 718 | 10 |
| Cyprus and Greece | 143 | 6 | 4 |
| Denmark | 400 | 3 | 1 |
| Egypt | 33 | 4 | 12 |
| Finland | 200 | 18 | 9 |
| France | 240 | 31 | 13 |
| Gaza-Palestine | 150 | 0 | 0 |
| Germany | 4649 | 73 | 2 |
| India | 4715 | 286 | 6 |
| Indonesia | 142 | 13 | 9 |
| Iran | 6733 | 449 | 7 |
| Ireland | 78 | 2 | 3 |
| Israel | 61 | 5 | 8 |
| Italy | 3291 | 118 | 4 |
| Japan | 342 | 40 | 12 |
| Jordan | 100 | 2 | 2 |
| Korea | 2572 | 243 | 9 |
| Kuwait | 266 | 7 | 3 |
| Latvia | 105 | 5 | 5 |
| Macedonia | 218 | 9 | 4 |
| Morocco | 83 | 16 | 19 |
| New Zealand | 125 | 7 | 6 |
| Pakistan | 89 | 4 | 4 |
| Russia | 810 | 61 | 8 |
| Saudi Arabia | 247 | 8 | 3 |
| Serbia | 326 | 29 | 9 |
| Singapore | 202 | 6 | 3 |
| Syria | 162 | 46 | 28 |
| Slovakia | 226 | 8 | 4 |
| Slovenia | 226 | 11 | 5 |
| Spain | 343 | 20 | 6 |
| Sri Lanka | 96 | 7 | 7 |
| Sweden | 192 | 4 | 2 |
| Taiwan | 180 | 19 | 11 |
| Tanzania | 49 | 0 | 0 |
| Thailand | 130 | 6 | 5 |
| Tunisia | 349 | 27 | 8 |
| Turkey | 875 | 67 | 8 |
| UK | 100 | 8 | 8 |
| **Total** | **40127** | **2717** | **7** |

**Table S1. Table showing prevalence of Yq microdeletions in different countries. Data for different countries were collected form PubMed.** Only those articles published in English were considered. The total number of infertile men studied and those having deletions were recorded along with the country. For each country data from different studies were pooled and the percentage estimated. Infertile men could be oligozoospermic or azoospermic men.

| **Sr No** | **Gene** | **Gene name** | **Location** | **Putative Functions** | **Cellular expression in testis** | **Method of detection** | **Expressed in other tissues** | **Role In Spermatogenesis** |
| --- | --- | --- | --- | --- | --- | --- | --- | --- |
| 1 | AMELY | Amelogenin, Y | Yp11.2 | Amelogenin family of extracellular matrix proteins | Weak RNA in testis | RNAseq | RNA in thyroid | Unknown |
| 2 | ASMT | Acetylserotonine O - methyltransferase | PAR1 | Catalyses final reaction in melatonin synthesis | NA | NA | RNA in epididymis | unknown |
| 3 | ASMTL | Acetylserotonine O -methyltransferase-like | PAR1 | methyltransferase activity | Germ and Leydig cells | IHC | Multiple | unknown |
| 4 | BPY1 and BPY1B | basic protein, Y1 | - Yq11.221   AZFa | encodes a protein that interacts with ubiquitin protein ligase E3A and may be involved in male germ cell development and male infertility | Both expressed in Spermatogonia,  preleptotene spermatocytes,  pachytene spermatocytes,  round and elongated spermatids | IHC | Testis specific | Unknown |
| 5 | BPY2, BPY2B and BPY2C | basic protein, Y2 | Yq11.223  BPY2 in AZFb/c (g1),  BPY2B in AZFb/c (g2)  and BPY2C in AZFc (g3) | Similar to BPY1 and BPY1B | RNA in testis | RNASeq | Testis specific | Regulation of the cyto-skeletal network |
| 6 | CDY1A and CDY1B | chromodomain, Y | Yq11.23  CDY1A in AZFb/c (yel1),  CDY1B in AZFb and AZFc (yel2) | encodes a protein containing a chromodomain and a histone acetyltransferase catalytic domain | Both genes RNA in testis | RNAseq | Testis specific | histone acetyltransferase catalytic domain |
| 7 | CDY2A | chromodomain, Y | Yq11.222  AZFb (yel3) | Similar to CDY1A and CDY1B | RNA in testis | RNAseq | Testis and epididymis | Gene expression regulation and post-meiotic nuclear remodelling |
| 8 | CRLF2 | cytokine receptor-like factor 2 | PAR1 | encodes a member of the type I cytokine receptor family | NA | NA | NA | NA |
| 9 | CSF2RA | colony-stimulating factor 2 receptor, alpha | PAR1 | protein encoded by this gene is the alpha subunit of the heterodimeric receptor for colony stimulating factor 2 | RNA in testis | RNASeq | Multiple tissues, highest in placenta | unknown |
| 10 | CSFR2Rα | GM-CSF receptor α subunit | PAR1 | Receptor that binds granulocyte-macrophage colony-stimulating factor | NA | NA | Multiple tissues  (RNA only) | unknown |
| 11 | CSPG4LY | Chondroitin Sulfate Proteoglycan 4 Pseudogene 1, Y-Linked | Yq11.23  AZFb and AZFc | Pseudogene | NA | NA | NA | unknown |
| 12 | CYorf15A  and CYorf15B | Chromosome Y  open reading frame  15 | Yq11.22  AZFb | Pseudogene | NA | NA | NA | unknown |
| 13 | DAZ1, DAZ2  and DAZ4 | Deleted in azoospermia | Yq11.223  DAZ1 in AZFb/c (r1),  DAZ2 in AZFb/c (r2)  DAZ4 in AZFc(r4) | encodes an RNA-binding protein that is important for spermatogenesis | Both expressed in Spermatogonia,  preleptotene and pachytene spermatocytes | IHC | RNA in testis and stomach  Protein exclusively in testis | Pre-meiotic regulation of transcript transport/storage, translation initiation and protein interaction |
| 14 | DAZ3 | Deleted in azoospermia | Yq11.223  AZFc(r3) | Similar to DAZ1, DAZ2 and DAZ3 | Expressed in Spermatogonia,  preleptotene and pachytene spermatocytes | IHC | Testis specific | Pre-meiotic regulation of transcript transport/storage, translation initiation and protein interaction |
| 15 | DBY | DEAD-box helicase 3, Y-linked | - Yq11.221   AZFa | encodes a protein that is a member of the DEAD-box RNA helicase family | Germ cells maximum in spermatogonia | IHC | Multiple tissues  RNA highest in testis and protein highest in tonsils | RNA metabolism in pre-meiotic germ cells (deduced by similarity) |
| 16 | EIF1AY | Eukaryotic Translation Initiation Factor 1A, Y-Linked | Yq11.223  AZFb | encodes a protein related to eukaryotic translation initiation factor 1A | Both in Germ and Leydig Cells | IHC | Multiple tissues | Regulation of translation initiation (deduced by similarity) |
| 17 | GOLGA1, GOLGA2LY, GOLGA3, GOLGA5, GOLGA7 | Golgi Autoantigen | Yq11.23  GOLGA2LY in AZFb and AZFc | gene is a member of the golgin gene family | Both in Germ and Leydig Cells | IHC | Multiple tissues | unknown |
| 18 | GOLGA6B and GOLGA6D | Golgi Autoantigen | Yq11.23 | gene is a member of the golgin gene family | Spermatogonia, round and elongated spermatids | IHC | RNA in liver and testis  Protein in testis only | unknown |
| 19 | GOLGA6C | Golgi Autoantigen | Yq11.23 | gene is a member of the golgin gene family | Spermatogonia, round and elongated spermatids | IHC | Testis specific | unknown |
| 20 | GOLGA6L7P | Golgi Autoantigen | Yq11.23 | gene is a member of the golgin gene family | RNA in testis | RNASeq | Testis specific | unknown |
| 21 | GTPBP6 | GTP binding protein 6 | PAR1 | encodes a GTP binding protein | Germ cells | IHC | Multiple tissues, highest in soft tissue | unknown |
| 22 | HSFY1 and  HSFY 2 | Heat shock factor, Y linked | Yq11.222  AZFb | encodes a member of the heat shock factor family of transcriptional activators for heat shock proteins | Both RNA expressed in testis | RNASeq | Testis specific | splicing regulator during spermatogenesis |
| 23 | HSPRY3 | Human-sprouty 3, Y linked | PAR2 | Pseudogene | NA | NA | NA | unknown |
| 24 | IL3RA | Interleukin-3 receptor α subunit | PAR1 | Alpha subunit for receptors for interleukin 3 | Germ and Leydig cells | IHC | Multiple tissues, highest in brain and fallopian tube | unknown |
| 25 | CD99 | CD99 Molecule  (Xg Blood Group) | PAR1 | Encodes a cell surface glycoprotein | Germ cells | IHC | Multiple tissues | unknown |
| 25 | NLGN4Y | Neuroligin 4, Y-linked | Yq11.221 | encodes a type I membrane protein that belongs to the family of cell adhesion molecules | RNA expressed in testis | RNASeq | RNA in multiple tissues  Protein exclusively in colon and brain | unknown |
| 26 | P2RY8 | purinergic receptor P2Y, G-protein coupled, Y linked 8 | PAR1 | protein encoded by this gene belongs to the family of G-protein coupled receptors | RNA in testis | RNASeq | Multiple tissues | unknown |
| 27 | PCDH11Y | Protocadherin 11, Y linked | Yp11.2 | Encodes a protein containing cadherin repeats | NA | RNASeq | RNA in cerebral cortex, placenta and seminal vesicles | unknown |
| 28 | PGPL | Pseudoautosomal GTP-binding protein-like | PAR1 | Encodes a GTP binding protein | Germ cells | IHC | Multiple tissues | unknown |
| 29 | PLCXD1 | Phosphatidylinositol specific phospholipase C, X domain containing 1 | PAR1 | unknown | Both in Germ and Leydig Cells | IHC | Multiple tissues | unknown |
| 30 | PPP2R3B | Protein phosphatase 2, regulatory subunit B | PAR1 | product of this gene belongs to the beta subfamily of regulatory subunit B | RNA in testis | RNASeq | Multiple tissues, highest in Skeletal Muscle | unknown |
| 31 | PRKY | Protein kinase, Y linked | Yp11.2 | Pseudogene | NA | NA | NA | unknown |
| 32 | PRY/PRY2 | putative tyrosine phosphatase protein-related Y | - Yq11.223   PRY in AZFb (b1) | low degree of similarity to protein tyrosine phosphatase, non-receptor type 13 | Weak RNA in testis | RNAseq | RNA in epididymis | Germ cell apoptosis |
| 33 | RBMY1A1 | RNA-binding motif, Y linked | - Yq11.223   AZFb | encodes a protein containing an RNA-binding motif in the N-terminus and four SRGY (serine, arginine, glycine, tyrosine) boxes in the C-terminus | Expressed in Spermatogonia,  preleptotene spermatocytes,  Pachytene spermatocytes,  Round spermatids | IHC | Testis specific | RNA splicing and metabolism, signal transduction and meiotic regulation |
| 34 | RPS4Y1 and RPS4Y2 | Ribosomal protein S4, Y linked | RPS4Y1 in Yp11.2  RPS4Y2 in Yq11.223 [AZFb] | Encode isoforms of ribosomal protein S4 | Both in Germ and Leydig Cells | IHC | RPS4Y1 in Multiple tissues  RPS4Y2 in testis and prostate only | Regulation of mRNA binding to the ribosome |
| 35 | SHOX | Short stature homeobox-containing | PAR1 | Transcription  factor associated to short stature | NA | NA | Multiple | unknown |
| 36 | SLC25A6 or ANT3, ANT3Y | Adenine nucleotide translocase or  Solute carrier family 25 member A6 | PAR1 | Gene product functions as a gated pore that translocates ADP and ATP | Germ and Leydig cells | IHC | Multiple | unknown |
| 37 | SMCY | Selected Mouse cDNA, Y | Yq11.223  AZFb | encodes a protein containing zinc finger domains | RNA in testis | RNASeq | RNA in Multiple tissues, highest in small intestine | Chromatin remodelling in meiosis |
| 38 | SRY | Sex Determining region, Y linked | Yp11.2 | testis-determining factor | RNA Germ cells and sperm  (see ref 37) | RNAseq and In situ hybridization | Predominantly in testis, low abundance RNA in skin and gastro-intestinal tract | initiates male sex determination and development of the male germ line |
| 39 | SYBL1 | Synaptobrevin-like 1 | PAR2 | Encodes a transmembrane protein that is a member of the soluble N-ethylmaleimide-sensitive factor attachment protein receptor family | Both in Germ and Leydig Cells | IHC | Multiple tissues | unknown |
| 40 | TBL1Y | Transducin beta like 1, Y-linked | Yp11.2 | protein encoded by this gene has sequence similarity with members of the WD40 repeat-containing protein family | RNA in testis and prostate | RNASeq | RNA highest in prostate and thyroid | unknown |
| 41 | TGIF2LY | TGFB induced factor homeobox 2 like, Y-linked | Yp11.2 | encodes a member of the TALE/TGIF homeobox family of transcription factors | RNA expressed in testis | RNASeq | Testis specific | transcriptional role in testis |
| 42 | TB4Y | Thymosin 4, Y isoform | Yq11.221  [AZFa] | encodes an actin sequestering protein | RNA in testis and prostate | RNAseq | RNA highest in rectum, testis and prostate | Unknown |
| 43 | TSPY1 | testis-specific protein, Y linked | Yp11.2 | protein encoded by this gene is found only in testicular tissue and may be involved in spermatogenesis | Spermatogonia and preleptotene spermatocytes | IHC | Testis specific | Spermatogonial proliferation in a phosphorylation-dependent manner; TSPY1 CNV affects susceptibility to spermatogenic failure by modulating the efficiency of spermatogenesis |
| 44 | TTTY1 | Testis transcript, Y1 | Yp11.2 | Not protein coding | NA | NA | NA | Unknown |
| 45 | TTTY2 | testis transcript, Y2 | Yp11.2 | Non protein coding | NA | NA | NA | Unknown |
| 46 | USP9Y | ubiquitin-specific protease, Y linked | Yq11.221  [AZFa] | encodes a protein that is similar to ubiquitin-specific proteases | RNA in testis | RNAseq | Multiple tissues with highest in prostate | Involved in protein turnover in spermatogenesis |
| 47 | UTY | Ubitiquitous TRY motif, Y linked | - Yq11.221   [AZFa] | encodes a protein containing tetratricopeptide repeats involved in protein-protein interactions | Low in germ cells | IHC | Multiple tissues  RNA highest in testis and protein highest in brain, spleen and tonsils | Mutations in this gene result in male infertility, a reduction in germ cell numbers, and can result in Sertoli-cell only syndrome |
| 48 | XE7 | X-escapee | PAR1 | Part of the spliceosome complex involved in the regulation of alternate splicing in some mRNA precursors | Germ cells high/Leydig cells low | IHC | Multiple tissues | unknown |
| 49 | XKRY | XK, Kell blood  group complex  subunit-related,  Y-linked | - Yq11.222   [AZFb  (yel3)] | encodes a protein which is a putative membrane transport protein | Both in Germ and Leydig Cells | IHC | Multiple tissues | Gamete interaction |
| 50 | ZBED1 | Zinc finger BED-type containing 1 | PAR1 | Encodes transcription factors that bind to DNA elements found in the promoter regions of several genes related to cell proliferation | RNA in testis | RNASeq | Multiple tissues | unknown |
| 51 | ZFY | Zinc-finger Y linked | Yp11.2 | encodes a zinc finger-containing protein that may function as a transcription factor | Germ and Leydig cells | IHC | Multiple tissues | appears to be involved in sperm or testis maturation |

**Table S2.** **Genes on the PAR1, PAR2 and NRY of the human Y chromosome.** Their location, putative functions, cellular expression in testis, method of detection, expression in other tissues, role in spermatogenesis. [Source <https://www.proteinatlas.org/> accessed on 31^st^ January 2018.]
